# Development and Characterization of Nanobody-Derived CD47 Theranostic Pairs in Solid Tumors

**DOI:** 10.34133/research.0077

**Published:** 2023-03-15

**Authors:** You Zhang, Di Zhang, Shuxian An, Qiufang Liu, Chenyi Liang, Juan Li, Ping Liu, Changfeng Wu, Gang Huang, Weijun Wei, Jianjun Liu

**Affiliations:** ^1^ Department of Nuclear Medicine, Institute of Clinical Nuclear Medicine, Renji Hospital, School of Medicine, Shanghai Jiao Tong University, Shanghai 200127, China.; ^2^ Department of Nuclear Medicine, Fudan University Shanghai Cancer Center, Fudan University, Shanghai 200030, China.; ^3^ Institute of Cancer and Basic Medicine, Chinese Academy of Sciences, The Cancer Hospital of the University of Chinese Academy of Sciences, Hangzhou 310022, Zhejiang, China.; ^4^ School of Biomedical Engineering and Med-X Research Institute, Shanghai Jiao Tong University, Shanghai 200030, China.; ^5^ Department of Biomedical Engineering, Southern University of Science and Technology, Shenzhen 518055, Guangdong, China.

## Abstract

Overexpression of CD47 is frequently observed in various types of human malignancies, inhibiting myeloid-mediated elimination of tumor cells and affecting the prognosis of cancer patients. By mapping biomarker expression, immuno-positron emission tomography has been increasingly used for patient screening and response monitoring. By immunization alpacas with recombinant human CD47, we prepared a CD47-targeting nanobody C2 and developed [^68^Ga]Ga-NOTA-C2, followed by an exploration of the diagnostic value in CD47-expressing tumor models including gastric-cancer patient-derived xenograft models. By fusing C2 to an albumin binding domain (ABD), we synthesized ABDC2, which had increased in vivo half-life and improved targeting properties. We further labeled ABDC2 with ^68^Ga/^89^Zr/^177^Lu to develop radionuclide theranostic pairs and evaluated the pharmacokinetics and theranostic efficacies of the agents in cell- and patient-derived models. Both C2 and ABDC2 specifically reacted with human CD47 with a high *K*_D_ value of 23.50 and 84.57 pM, respectively. [^68^Ga]Ga-NOTA-C2 was developed with high radiochemical purity (99 >%, *n* = 4) and visualized CD47 expression in the tumors. In comparison to the rapid renal clearance and short half-life of [^68^Ga]Ga-NOTA-C2, both [^68^Ga]Ga-NOTA-ABDC2 and [^89^Zr]Zr-DFO-ABDC2 showed prolonged circulation and increased tumor uptake, with the highest uptake of [^89^Zr]Zr-DFO-ABDC2 occurring at 72 h post-injection. Moreover, [^177^Lu]Lu-DOTA-ABDC2 radioimmunotherapy suppressed the tumor growth but was associated with toxicity, warranting further optimization of the treatment schedules. Taken together, we reported a series of nanobody-derived CD47-targeted agents, of which [^68^Ga]Ga-NOTA-C2 and [^89^Zr]Zr-DFO-ABDC2 are readily translatable. Optimization and translation of CD47-targeted theranostic pair may provide new prospects for CD47-targeted management of solid tumors.

## Introduction

The cluster of differentiation 47 (CD47) is the only known 5-transmembrane receptor member of the immune system which widely expressed across different cell types in the body [1]. It plays a physiological role by binding to signal regulatory protein α (SIRPα), affecting the maintenance of red blood cells, platelets, and hematopoietic stem cells, and regulates synaptic pruning during neuron development [2]. However, tumor cells hijack this mechanism conveying a “don’t eat me” signal to macrophages to evade the clearance by macrophages [3,4]. Overexpression of CD47 is correlated with poor prognosis in a plethora of malignancies, such as acute myeloid leukemia [5], colorectal cancer [6], and lymphoma [7], among others. Preclinical studies have verified the therapeutic effect of anti-CD47–SIRPα axis therapy [8–14]. Moreover, the anti-CD47 antibody magrolimab combined with rituximab had a confirmative therapeutic effect in non-Hodgkin’s lymphoma [15]. Its therapeutic role in patients with other types of advanced cancers was further confirmed in 2019 [16]. Inhibiting CD47 is a promising cancer therapeutic strategy. However, the widespread expression of CD47 in normal tissues acts as an antigen sink and inevitably results in the deposition of the administered antibodies in normal tissues. An increased administered dose is needed to achieve an antitumor effect but is associated with side effects [2,15]. Therefore, there is an urgent demand for developing companion diagnostic tools to help stratify patients who may benefit from anti-CD47 therapies. Besides developing companion diagnostic tools, innovating CD47-targeted therapeutics may further enrich the CD47-targeted theranostic toolbox.

By exquisitely fusing the extraordinary targeting specificity of the antibody and the superior sensitivity of positron emission tomography (PET), immuno-positron emission tomography (immunoPET) can noninvasively display the expression of targets of interest across the body [17]. For instance, an immunoPET imaging probe targeting programmed cell death ligand-1 (PD-L1) has been successfully applied to clinical practice. More importantly, PD-L1 level quantified by immunoPET, but not revealed by immunohistochemistry (IHC), predicted the response of atezolizumab [18]. In addition, immunoPET probes can evaluate the dynamics of lymphocytes and myeloid cells before and after immunotherapies and further reveal the immunity status inside the tumors [19–21]. On the basis of this evidence and our previous findings [22,23], we hypothesize that CD47-targeted immunoPET can noninvasively display heterogeneous CD47 inside the tumors and select patients suitable for CD47-targeted immunotherapies. Meanwhile, accumulating evidence suggests that radioimmunotherapy (RIT) and pretargeted RIT may halt tumor growth [24,25] and even eradicate certain types of cancer [26–28].

There are not any CD47-targeted theranostic pairs reported so far. Zheleznyak et al. [29] developed an ^89^Zr-labeled monoclonal antibody (mAb) tracer against CD47 and demonstrated the feasibility of the tracer in displaying the CD47 expression inside the tumors. However, the application of radiolabeled mAbs is hampered by high cost, the necessity to use long-lived radionuclides, cumbersome imaging processes across a week, and the associated radiation exposure to the patients and staff. To accelerate the application of antibody diagnostics in the clinic, pretargeted imaging strategies or the use of smaller antibody fragments or antibody mimetics that facilitate same-day molecular imaging is actively explored [30]. Of these smaller vectors, nanobody or single-domain antibody derived from *Camelidae* is the smallest antigen-binding unit with a molecular weight of around 15 kDa. The small size, high affinity, and ease of engineering make nanobodies powerful alternatives for molecular imaging [31,32]. In recent years, we are focusing on the development and clinical translation of nanobody-derived tracers for their superior molecular imaging traits [33–35]. Radiolabeled monovalent nanobodies are ideal as companion diagnostic tools, but the in vivo half-lives are too short and kidney accumulation is exceptionally high, leaving room for further improvement. To develop an integrated theranostic platform, albumin binding domain (ABD) targeting human/murine albumin was introduced into nanobodies to prolong the in vivo half-lives of nanobody derivatives [36]. We and others have reported that bispecific nanobody derivatives simultaneously targeting tumor antigens and albumin have improved biodistribution profiles and can serve as vectors for developing the theranostic toolbox [37,38].

To fill in the gap in the field, we herein describe the development of nanobody-derived CD47-targeting theranostic pairs and characterize the theranostic value in cell-derived xenograft and patient-derived xenograft (PDX) models.

## Results

### Detection of CD47 expression on different tumor cells

**Fig. 1.**
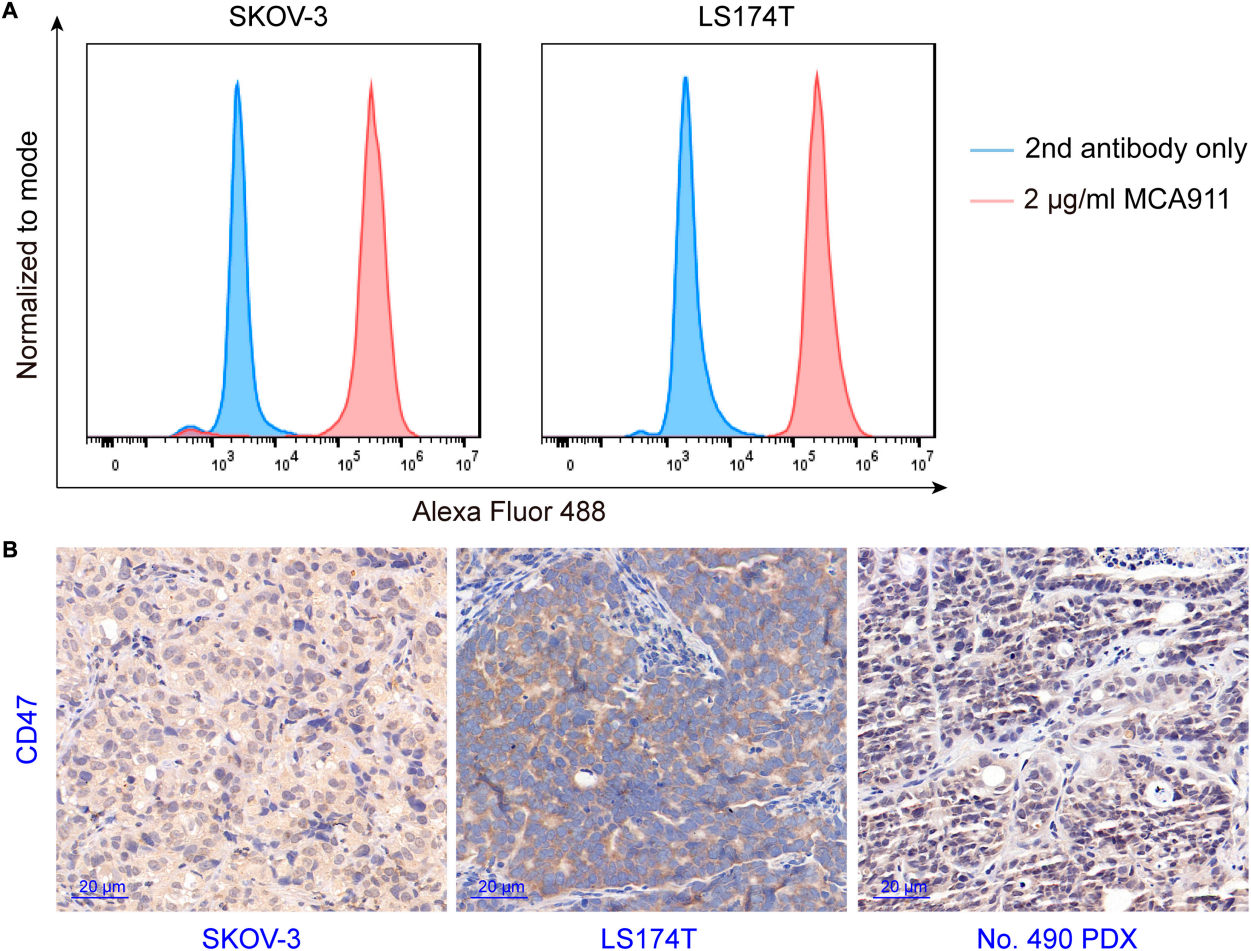
CD47 was highly expressed on SKOV-3 ovarian and LS174T colorectal cancer cells, as well as in the No. 490 gastric cancer PDX models. (A) Flow cytometry histogram of SKOV-3 and LS174T cells incubated with 2 μg/ml MCA-911 and a second antibody, or second antibody only. (B) Immunohistochemical images of 3 representative CD47-positive tumors. B6H12 was used as the primary antibody for staining.

MCA911 was used as the primary antibody to detect the expression of CD47 on different cancer cell lines. As shown in Fig. 1A, CD47 was highly expressed in the SKOV-3 (an ovary cancer cell line) and LS174T (a colorectal cancer cell line) cells. We further performed IHC staining of 9 archived tumors with an immunoglobulin G1 (IgG1) κ mAb (B6H12) (Fig. 1B and Fig. S1). On the basis of the flow cytometry and IHC scouting results, we selected SKOV-3, LS174T, and a PDX (No. 490 gastric cancer) models with relatively high CD47 expression for the experiments.

### Determination of in vitro binding affinities

**Fig. 2.**
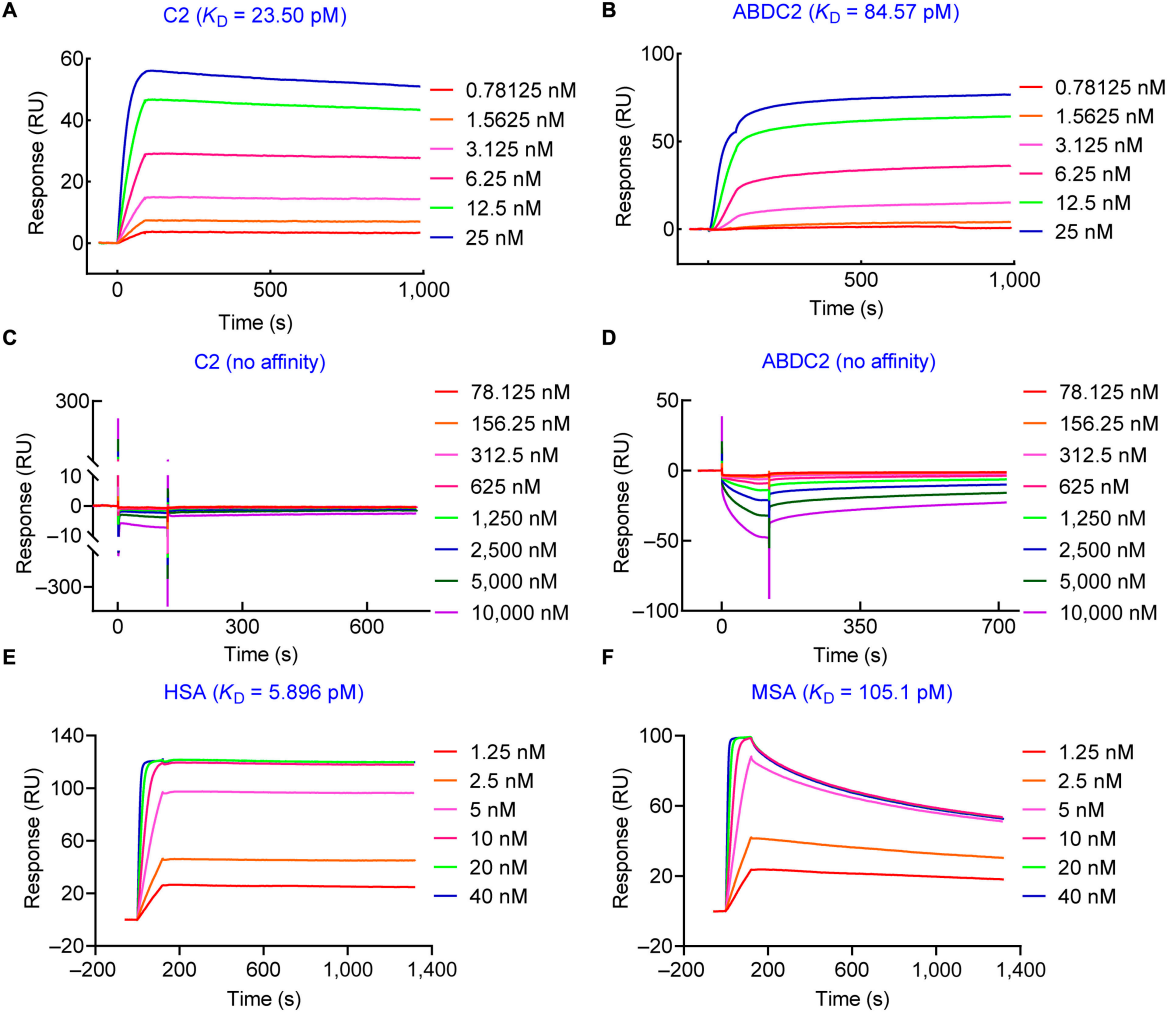
Surface plasmon resonance studies showing the association and dissociation kinetics of C2 (A and C) and ABDC2 (B and D) interacting with recombinant human (A and B) and mouse (C and D) CD47 proteins. ABDC2 also showed high binding affinities to human serum albumin (HSA) (E) and murine serum albumin (MSA) (F). RU, response units.

We primarily selected 3 clones for the current study. C1 and C3 were 2 clones obtained from immunization of Bactrian camel but were poorly soluble when expressed in *E. coli* (Figs. S2 and S3). Specifically, C1 was expressed at a level of 11.36 mg/l, but 1 M L-arginine was added to the buffer because of the poor solubility (Fig. S4). We assume that the existence of 2 pairs of disulfide bonds contributed to the poor solubility. In comparison, C2 has 1 pair of disulfide bonds and was highly soluble in phosphate buffer saline (PBS) and expressed at a level of 39.1 to 44.70 mg/l (Fig. S5). ABDC2, a nanobody derivative consisting of C2 and albumin binder ABD035 with a flexible (GGGGS)_3_ linker, was expressed a high yield of 80.7 mg/l. The purities of C2 and ABDC2 were cross-validated as shown in Fig. S6. Therefore, C2 and ABDC2 were selected for subsequent imaging and therapy studies. Both the monovalent nanobody C2 and the fused protein ABDC2 could selectively bind to recombinant human CD47 with excellent *K*_D_ values of 23.50 and 84.57 pM, respectively (Fig. 2A and B). The ABD035 sequence introduced in ABDC2 did not noticeably weaken its affinity with the human CD47 antigen. Of interest, neither C2 nor ABDC2 had an affinity with mouse CD47 (Fig. 2C and D). Besides, ABDC2 has a high affinity with human serum albumin and murine serum albumin, with *K*_D_ values of 5.896 and 105.1 pM, respectively (Fig. 2E and F). As expected, random chelator conjugation of C2 and ABDC2 impaired the binding affinities, but the measured *K*_D_ values were still within low picomoles. The calculated *K*_D_ value of NOTA-C2, NOTA-ABDC2, DOTA-C2, DOTA-ABDC2, and DFO-ABDC2 was 137.5, 204.3, 189.1, 260.8, and 275.1 pM, respectively (Fig. S7). The robust data suggest that C2 and ABDC2 are potent candidates for developing molecular imaging or theranostic agents.

### Development and characterization of radiotracers

To design immunoPET imaging probes targeting CD47, the chelator-modified C2 and ABDC2 were labeled with ^68^Ga (*T_1/2_* = 1.1 h) or ^89^Zr (*T_1/2_* = 78.4 h), respectively. The calculated NOTA/C2 ratio was 1.3 for NOTA-C2, and the ratio for NOTA-ABDC2 was 6.6 (Fig. S8). The nondecay corrected radiochemical yield of [^68^Ga]Ga-NOTA-C2, [^68^Ga]Ga-NOTA-ABDC2, and [^89^Zr]Zr-DFO-ABDC2 were 25.15% ± 10.21 (*n* = 4), 12.85% ± 0.45 (*n* = 2), and 99% (*n* = 1), respectively. The radiochemical purity (RCP) of all the radiotracers were >99% as assessed by instant thin-layer chromatography (Figs. S9 and S10), indicating the developed tracers meet the standards for in vivo molecular imaging. The nondecay corrected radiolabeling yield of [^177^Lu]Lu-DOTA-C2 and [^177^Lu]Lu-DOTA-ABDC2 was 35.41% (*n* = 1) and 62.76% (*n* = 1), respectively. The RCP of [^177^Lu]Lu-DOTA-C2 and [^177^Lu]Lu-DOTA-ABDC2 remained 52% and 72% at 72 h in PBS (Fig. S11).

### Pharmacokinetics of [^68^Ga]Ga-NOTA-C2 and [^68^Ga]Ga-NOTA-ABDC2 in tumor-free mice

**Fig. 3.**
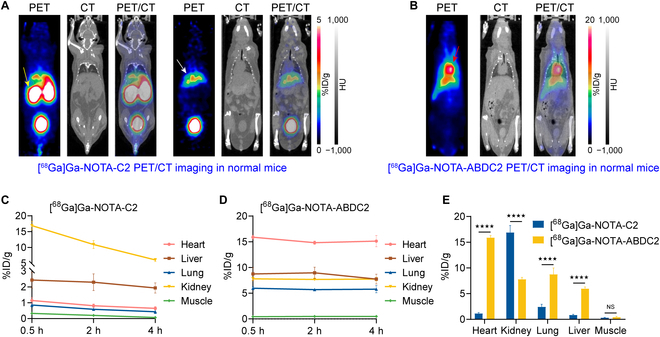
Pharmacokinetic studies of [^68^Ga]Ga-NOTA-C2 and [^68^Ga]Ga-NOTA-ABDC2 in tumor-free Balb/c mice. (A) [^68^Ga]Ga-NOTA-C2 immunoPET imaging in Balb/c mice at 0.5 h after injection of the tracer (6.98 ± 1.38 MBq, *n* = 3). Coronal images at different slices showed clear delineation of the kidneys (yellow arrows) and liver (white arrow). (B) [^68^Ga]Ga-NOTA-ABDC2 immunoPET imaging in tumor-free Balb/c mice at 0.5 h after injection of the tracer (5.75 ± 0.29 MBq, *n* = 4). Coronal images showed intensive accumulation in the heart (red arrow). (C and D) ROI data showing the kinetics of [^68^Ga]Ga-NOTA-C2 (C, *n* = 3) and [^68^Ga]Ga-NOTA-ABDC2 (D, *n* = 4) at different time points. (E) Head-to-head comparison of the ROI data at 0.5 h after injection of the tracers. ★★★★: *P* < 0.0001; NS, no statistical significance. HU, Hounsfield units.

After successful synthesis of the [^68^Ga]Ga-NOTA-C2 and [^68^Ga]Ga-NOTA-ABDC2, we first visualized them in tumor-free Balb/c mice to identify the in vivo pharmacokinetics. PET/computed tomography (CT) imaging was performed at 0.5 h (Fig. 3A and B), 2 h, and 4 h after injection of the tracers (Fig. S12). [^68^Ga]Ga-NOTA-C2 was rapidly excreted through kidneys into the bladder because of the small molecular weight (Fig. 3A), with small amount of uptake in the liver, heart, and lung (Fig. 3C). Consistent with the description in the literature [37,38], the introduction of albumin binder into ABDC2 significantly prolonged the in vivo circulation time of the probe (Fig. 3B). Regions of interest (ROI) analysis showed that accumulation in the heart did not decrease significantly at 4 h (15.13 ±1.09%ID/g, *n* = 3; Fig. 3D). Head-to-head comparison showed that heart retention of [^68^Ga]Ga-NOTA-ABDC2 (15.90 ± 0.39%ID/g, *n* = 3) was statistically higher than that (1.14 ± 0.19%ID/g, *n* = 3; *P* < 0.0001, Fig. 3E) of [^68^Ga]Ga-NOTA-C2 at 0.5 h. However, the kidney accumulation of [^68^Ga]Ga-NOTA-ABDC2 (7.80 ± 0.37%ID/g, *n* = 3) was much lower than that (16.90 ± 1.35%ID/g, *n* = 3) of [^68^Ga]Ga-NOTA-C2. The prolonged circulation of [^68^Ga]Ga-NOTA-ABDC2 also led to increased uptake in the lung and liver, both statistically different from that of [^68^Ga]Ga-NOTA-C2 (*P* < 0.0001). Collectively, ABD035 incorporation resulted in a 13.9-fold increase in blood circulation and a 2.2-fold decrease in kidney accumulation, making ABDC2 potentially useful for radionuclide therapy or nanobody–drug conjugate therapy.

### [^68^Ga]Ga-NOTA-C2 immunoPET imaging in cell-derived xenograft and PDX models

**Fig. 4.**
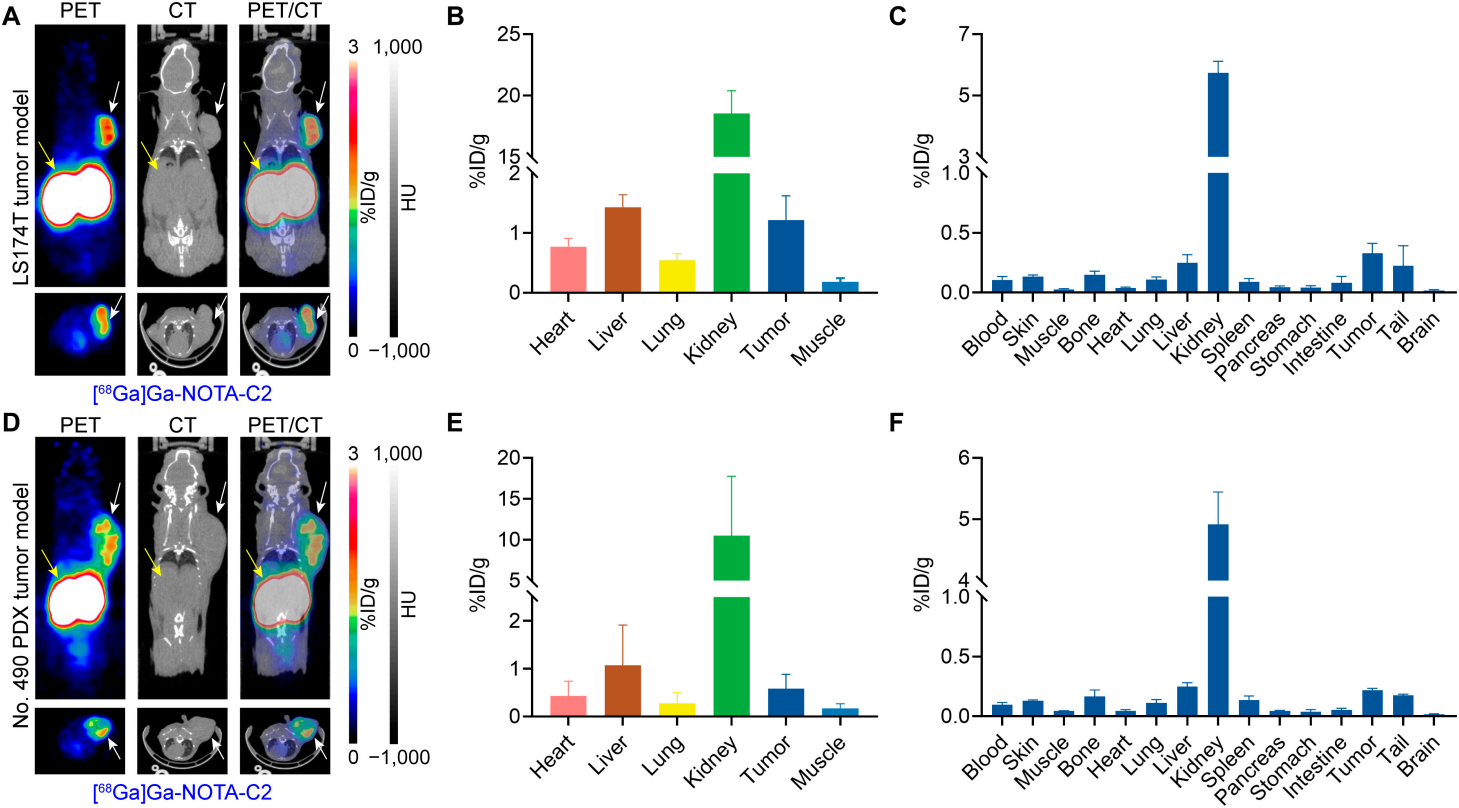
[^68^Ga]Ga-NOTA-C2 immunoPET imaging in cell- and patient-derived models. (A and D) [^68^Ga]Ga-NOTA-C2 immunoPET imaging in subcutaneous LS174T (A, 9.10 ± 0.89 MBq, *n* = 3) and No. 490 PDX (D, 6.50 ± 0.46 MBq, *n* = 3) models 1 h after tracer injection. Coronal (top panels) and axial (bottom panels) images showed clear delineation of the tumors (white arrows) and kidneys (yellow arrows). (B and E) ROI data showing the uptake kinetics of the tracer in LS174T (B) and No. 490 PDX (E) models. (C and F) The radioactive biodistribution data shows the accumulation of the tracer in LS174T (C) and No. 490 PDX (F) models.

ImmunoPET imaging with [^68^Ga]Ga-NOTA-C2 was then carried out in the subcutaneous LS174T (Fig. 4A to C) and No. 490 PDX (Fig. 4D to F) models. The results demonstrated that tumor uptake of [^68^Ga]Ga-NOTA-C2 was sharp in the 2 immunocompromised tumor models. A biodistribution study was carried out after the PET/CT scans to quantify the uptake of the tracer among various organs. As revealed by the ROI analysis (Fig. 4B and E), the average tumor uptake in the LS174T and No. 490 PDX models was 1.22 ± 0.33%ID/g (*n* = 3) and 0.58±0.24%ID/g (*n* = 3), respectively. There was no significant difference in tumor uptake values between the 2 models (*P* = 0.09, Fig. S13). The biodistribution data is consistent with the imaging results, showing tumor uptake of 0.33 ± 0.07%ID/g (*n* = 3, Fig. 4C) in LS174T models and 0.22 ± 0.01%ID/g (*n* = 3, Fig. 4F) in No. 490 PDX models, respectively. IHC staining results further confirmed the comparable CD47 expression in the 2 tumor models (Fig. S14B to D).

### Optimized [^68^Ga]Ga-NOTA-C2 immunoPET imaging in ovary cancer models

High kidney accumulation of a radioligand may not compromise the diagnostic efficacy; it affects the detection of urinary tumors and further causes unexpected nephrotoxicity when a therapeutic dose is administered [39]. The high kidney accumulation of [^68^Ga]Ga-NOTA-C2 largely resided in the renal cortex. Coinjection of sodium maleate could reduce kidney accumulation of nanobody-derived tracers [34]. Therefore, we optimized [^68^Ga]Ga-NOTA-C2 immunoPET imaging by this method (Fig. S15A to C). As shown by the quantitative analysis results (Fig. S15D), the kidney accumulation (5.45 ± 1.08%ID/g, *n* = 4) in the maleate intervention group was much lower than that (12.75±1.32%ID/g, *n* = 4; *P* < 0.0001) in the control group. Notably, there was no statistical difference in tumor uptake in the 2 groups.

### [^68^Ga]Ga-NOTA-ABDC2 immunoPET imaging in No. 490 PDX models

**Fig. 5.**
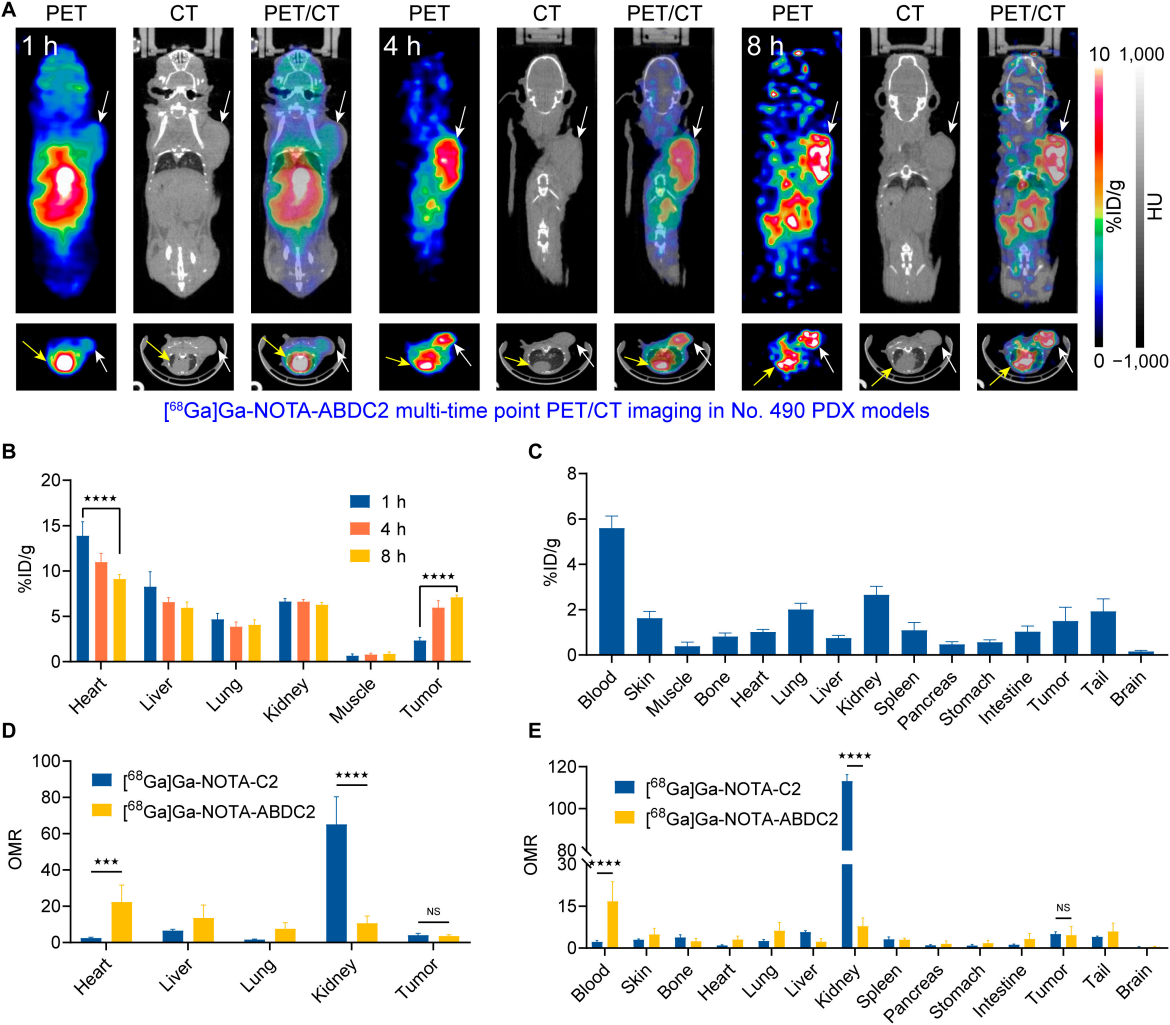
[^68^Ga]Ga-NOTA-ABDC2 immunoPET imaging in No. 490 gastric cancer PDX models (4.31 ± 0.22 MBq, *n* = 4). (A) Representative coronal (top panels) and axial (bottom panels) images of [^68^Ga]Ga-NOTA-ABDC2 immunoPET/CT in the PDX models. The images showed clear delineation of the tumors (white arrows) and hearts (yellow arrows). (B) ROI data of [^68^Ga]Ga-NOTA-ABDC2 immunoPET imaging at different time points. (C) Ex vivo biodistribution data showing the detailed uptake of [^68^Ga]Ga-NOTA-ABDC2 in the tumor, blood, major organs, and tissues. (D) Analysis of organ-to-muscle ratios (OMRs) at 1 h post-injection of the tracers. (E) Head-to-head comparison of the ex vivo biodistribution data in terms of organ-to-muscle ratios. ★★★★: *P* < 0.0001. ★★★: *P* < 0.0005.

Next, the targeting efficiency of [^68^Ga]Ga-NOTA-ABDC2 was investigated in the LS174T and PDX No. 490 models. We extended the imaging time to 8 h and performed imaging at 3 time points (i.e., 1, 4, and 8 h; Fig. 5A). Analysis of the ROI data showed a gradual increase in tumor uptake as the time elapses (Fig. 5B). The average tumor uptake at 1, 4, and 8 h was 2.35 ± 0.26%ID/g (*n* = 4), 5.98 ± 0.58%ID/g (*n* = 4), and 7.10 ± 0.20%ID/g (*n* = 4), respectively. The heart retention showed a gradual but slow decrease from 13.88 ± 1.21%ID/g (*n* = 4) at 1 h to 9.13 ± 0.39%ID/g (*n* = 4) at 8 h. Both the tumor uptake and the heart accumulation were significantly different at the 2 time points (*P* < 0.001). Meanwhile, the biodistribution of the tracer among various organs and tissues was defined after the PET/CT scans. As shown in the Fig. 5C, retention of the tracer in the blood pool (5.60 ± 0.46%ID/g, *n* = 4) was higher than uptake of the tracer in tumor (1.50 ± 0.53%ID/g, *n* = 4) at 8 h post-injection. Analysis of the ROI data at 1 h (Fig. 5D) showed that the heart-to-muscle ratio of [^68^Ga]Ga-NOTA-ABDC2 was significantly higher than that of [^68^Ga]Ga-NOTA-C2 (*P* < 0.0005), while the kidney-to-muscle ratio of [^68^Ga]Ga-NOTA-C2 is remarkably higher than that of [^68^Ga]Ga-NOTA-ABDC2 (*P* < 0.0001). The biodistribution data (Fig. 5E) further confirmed the above ROI analysis results. Furthermore, there is no difference in the tumor-to-muscle ratios (TMRs) between [^68^Ga]Ga-NOTA-C2 and [^68^Ga]Ga-NOTA-ABDC2 at 1 h post-injection. The following IHC staining of the collected tumors (Fig. S13D) confirmed CD47 expression. Taken together, we could conclude that [^68^Ga]Ga-NOTA-ABDC2 had significantly increased circulation time and improved pharmacokinetics for tumor imaging. However, we need to exclude the enhanced permeation and retention effect attributed to albumin hitchhiking. Furthermore, the short half-life of ^68^Ga limited a thorough evaluation of the tracer.

### [^68^Ga]Ga-NOTA-C2 and [^68^Ga]Ga-NOTA-ABDC2 blocking studies

**Fig. 6.**
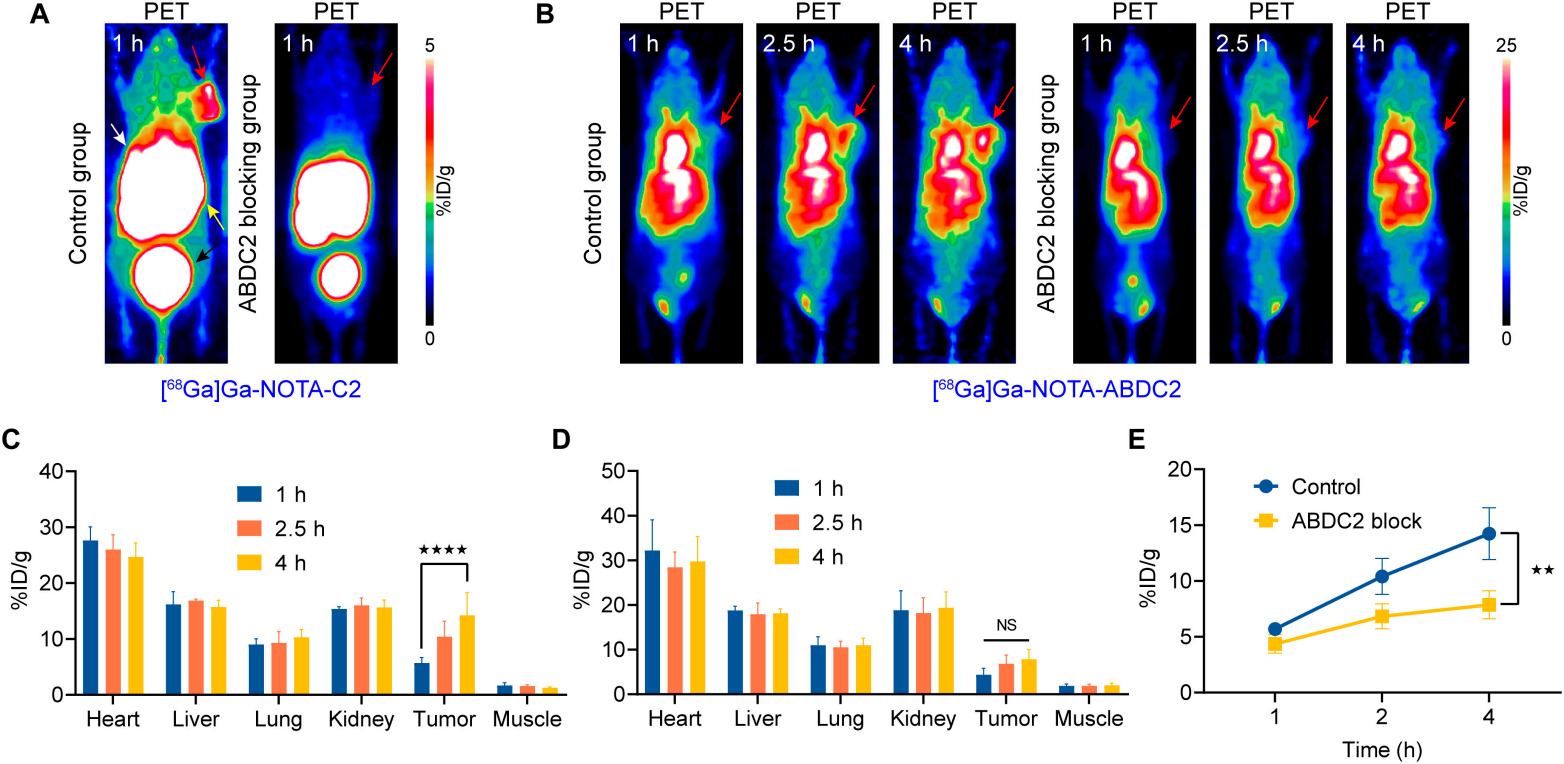
[^68^Ga]Ga-NOTA-C2 and [^68^Ga]Ga-NOTA-ABDC2 blocking studies in LS174T models. (A) [^68^Ga]Ga-NOTA-C2 immunoPET imaging in the control group and ABDC2 blocking group. (B) [^68^Ga]Ga-NOTA-ABDC2 immunoPET imaging in the control group and ABDC2 blocking group. Tumors (red arrows), livers (white arrow), kidneys (yellow arrow), and bladders (black arrow). Quantitative ROI analysis results in the control group ([^68^Ga]Ga-NOTA-ABDC2, C) and ABDC2 blocking group ([^68^Ga]Ga-NOTA-ABDC2 + ABDC2 premedication, D). (E) Comparison of tumor uptake of [^68^Ga]Ga-NOTA-ABDC2 at different time points between the control and ABDC2 blocking groups. ★★★★: *P* < 0.0001. ★★: *P* < 0.005.

The targeting specificities of [^68^Ga]Ga-NOTA-C2 and [^68^Ga]Ga-NOTA-ABDC2 were further investigated in the LS174T models. We divided the mice into 4 groups (*n* = 3 for each group): [^68^Ga]Ga-NOTA-C2 group, [^68^Ga]Ga-NOTA-C2 ABDC2 blocking group, [^68^Ga]Ga-NOTA-ABDC2 group, and [^68^Ga]Ga-NOTA-ABDC2 ABDC2 blocking group. Mice in the first 2 groups were imaged at 1 h, and mice in the latter 2 groups were imaged at 1, 2.5, and 4 h post-injection of the tracers. As can be seen from the PET images, tumor uptake in the control group was significantly higher than that in the ABDC2 blocking group (Fig. 6A and B). More specifically, the tumor uptake of [^68^Ga]Ga-NOTA-ABDC2 in the control group, but not the ABDC2 blocking group, gradually increased over time (Fig. 6C to E). It can be seen that there was a small amount of tumor uptake in the ABDC2 blocking group, indicating enhanced permeation and retention effect-mediated tumor trapping of [^68^Ga]Ga-NOTA-ABDC2. However, the uptake value is lower than that of the control group (Fig. 6E). The results demonstrate that both [^68^Ga]Ga-NOTA-C2 and [^68^Ga]Ga-NOTA-ABDC2 have high specificities for CD47 imaging.

### [^89^Zr]Zr-DFO-ABDC2 immunoPET imaging in No. 490 PDX models

**Fig. 7.**
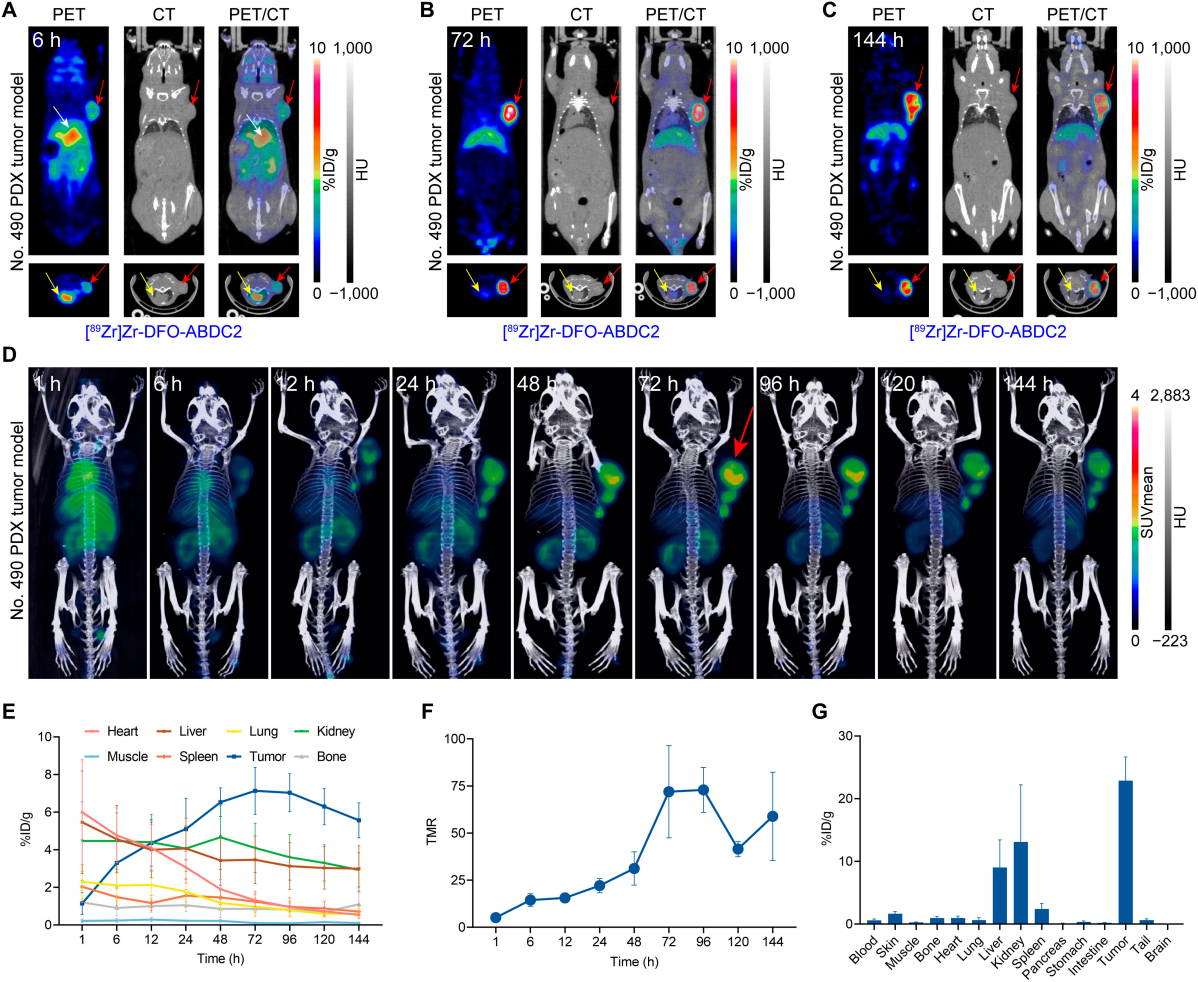
[^89^Zr]Zr-DFO-ABDC2 immunoPET imaging in No. 490 gastric cancer PDX models (4.10 ± 0.50 MBq, *n* = 3). Representative coronal (top panels) and axial (bottom panels) [^89^Zr]Zr-DFO-ABDC2 immunoPET/CT images at 6 h (A), 72 h (B), and 144 h (C). The tumors, hearts, and abdominal aortas were indicated by red arrows, yellow arrows, and white arrows, respectively. (D) The maximum intensity projection images fused with CT images of all the time points show the distribution patterns of [^89^Zr]Zr-DFO-ABDC2. (E) ROI data showing the uptake kinetics of [^89^Zr]Zr-DFO-ABDC2 at different time points. (F) Tumor-to-muscle ratio (TMR) analysis at different time points after injection of the tracer. (G) Ex vivo biodistribution data showing the detailed uptake and distribution of [^89^Zr]Zr-DFO-ABDC2.

[^68^Ga]Ga-NOTA-ABDC2 cannot accurately show the complete pharmacokinetics of ABDC2 because of its significantly prolonged serum half-life, so we used the long half-life radionuclide ^89^Zr for further imaging experiments. [^89^Zr]Zr-DFO-ABDC2 was developed with good radiochemical yield and RCP (Fig. S9). We performed imaging at 1, 6, 12, 24, 48, 72, 96, 120, and 144 h after the injection of the tracer. As shown in Fig. 7A to C, PET/CT images acquired at 6, 72, and 144 h displayed the uptake of the tracer in the tumor and also in the liver where it was catabolized. The maximum intensity projection images fused with CT images of all the time points fairly showed the overall distribution and uptake patterns of the tracer across a week (Fig. 7D and Fig. S16A). As shown by the ROI analysis data (Fig. 7E), tumor uptake gradually increased, peaked at 72 h (7.13 ± 1.02%ID/g, *n* = 3), and then gradually decreased to 5.57 ± 0.76%ID/g (*n* = 3) at 144 h, which was still higher than the uptake by other normal organs and tissues. Uptake in the heart, lung, liver, spleen, and kidneys showed a gradual decrease from 1 to 144 h. In addition, bone uptake did not increase until 144 h, demonstrating the stability of the probe and the absence of free zirconium in the circulation. TMR analysis was consistent with the ROI results (Fig. 7F), showing the plateau at 72 h. Biodistribution data showed that tumor uptake was significantly higher than that of the heart, liver, and kidneys (Fig. 7G). The following immunohistochemistry staining of the resected tumors confirmed CD47 expression in the No. 490 PDX models (Fig. S16B).

### Exploratory [^177^Lu]Lu-DOTA-ABDC2 theranostics in gastric cancer PDX models

**Fig. 8.**
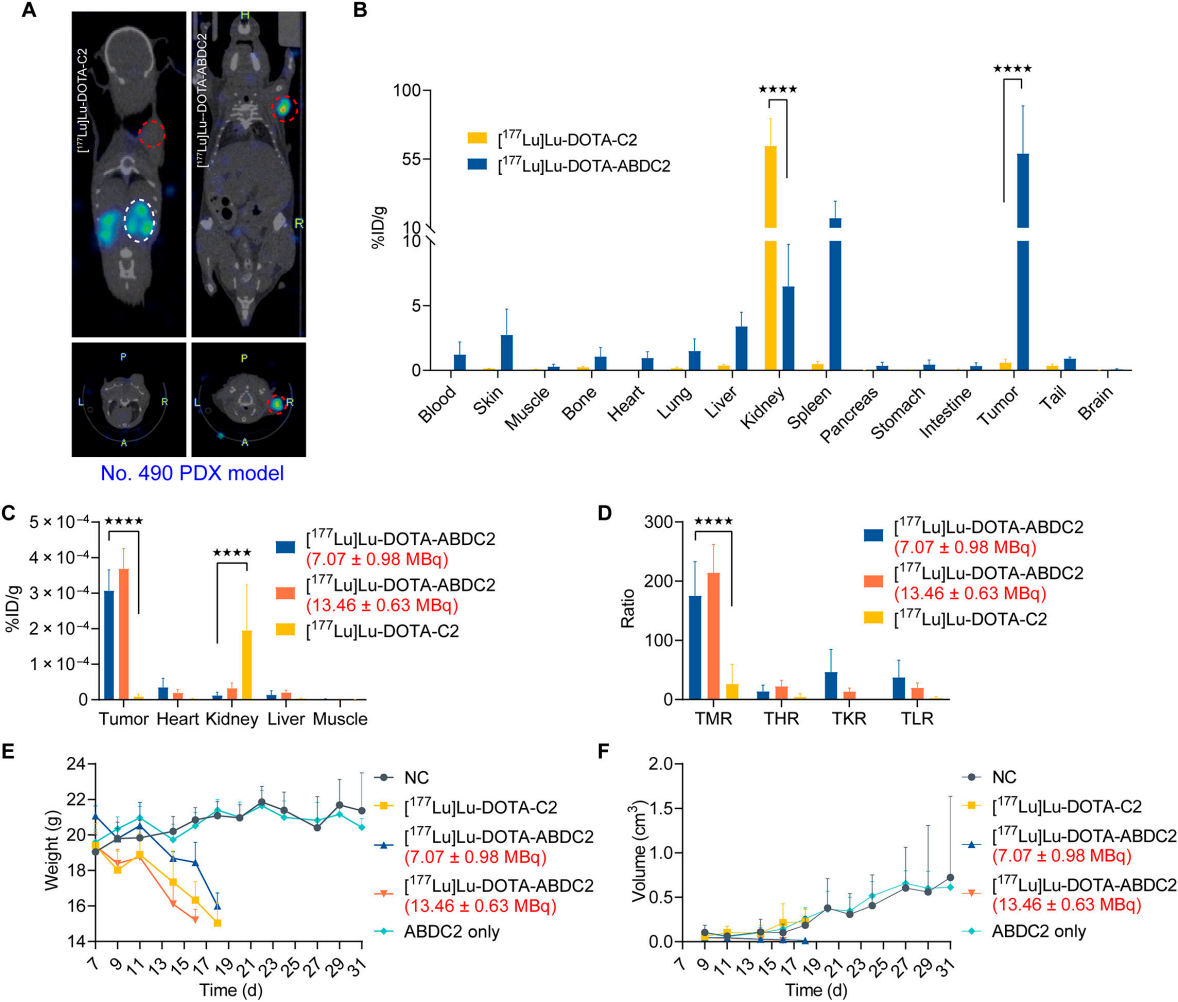
Initial [^177^Lu]Lu-DOTA-ABDC2 theranostics in gastric-cancer PDX models. (A) Representative coronal (top panels) and axial (bottom panels) [^177^Lu]Lu-DOTA-C2 and [^177^Lu]Lu-DOTA-ABDC2 immunoSPECT/CT images in No. 490 gastric cancer PDX models. [^177^Lu]Lu-DOTA-ABDC2 delineated the tumors (red dashed coil), while [^177^Lu]Lu-DOTA-C2 only displayed the kidneys (white dashed coil). (B) Biodistribution data was collected after [^177^Lu]Lu-DOTA-C2 and [^177^Lu]Lu-DOTA-ABDC2 immunoSPECT/CT imaging. (C) Quantitative uptake of the 3 tracers. (D) Analysis of the tumor-to-muscle ratio (TMR), tumor-to-heart ratio (THR), tumor-to-kidney ratio (TKR), and tumor-to-liver ratio (TLR) of the tracers. Curves show the changes in mouse body weights (E) and tumor volumes (F) over time. ★★★★: *P* < 0.0001.

To initially evaluate the effectiveness of ABDC2 as a radionuclide therapeutic vector, we incorporated ^177^Lu for treatment experiments. Mice bearing No. 490 PDX xenografts implanted 1 week earlier were divided into 5 groups. Specifically, the normal control group received an intravenous injection of 200 μl of PBS, [^177^Lu]Lu-DOTA-C2 treatment group received a single-dose intravenous injection of 200-μl [^177^Lu]Lu-DOTA-C2 (6.66 ± 0.80 MBq). Two different doses of [^177^Lu]Lu-DOTA-ABDC2, a low dose of 7.07 ± 0.98 MBq and a high dose of 13.46 ± 0.63 MBq, were scheduled. Moreover, the ABDC2 treatment group received an injection of 500 μg of ABDC2 in 200 μl of PBS (Table S2). The average body weight of all the mice was 19.69 ± 1.03 g (*n* = 26). We performed single photon emission computed tomography (SPECT)/CT imaging 1 week after the onset of the treatment (Fig. 8A). While tumor uptake of [^177^Lu]Lu-DOTA-C2 was negligible, tumor uptake of [^177^Lu]Lu-DOTA-ABDC2 was obvious. As shown in Fig. 8B, the tumor uptake of [^177^Lu]Lu-DOTA-ABDC2 was significantly higher than that of [^177^Lu]Lu-DOTA-C2. In comparison, the kidney retention of [^177^Lu]Lu-DOTA-ABDC2 was significantly lower than that of [^177^Lu]Lu-DOTA-C2 (*P* < 0.0001). The quantitative uptake of these 3 tracers was shown in Fig. 8C. The TMR, tumor-to-heart ratio, tumor-to-kidney ratio, and tumor-to-liver ratio were shown in Fig. 8D. The weight and tumor volume changes were shown in Fig. 8E and F, respectively. Unexpectedly, the mice in the treatment group successively died after SPECT/CT imaging, probably because of the high ^177^Lu dosage. Tumors in the [^177^Lu]Lu-DOTA-ABDC2 treatment groups were gradually eradicated, while tumor volume in the other 3 groups gradually and steadily increased. ABDC2 alone did show any therapeutic effect compared to the normal control group. These data suggest that while [^177^Lu]Lu-DOTA-ABDC2 had a therapeutic effect, the treatment schedules and doses need to be optimized.

## Discussion

CD47–SIRPα axis is the first tumor phagocytosis checkpoint pathway identified in the late 2000s. After that, the molecular mechanisms underlying the role of the CD47–SIRPα axis in normal and tumor tissues are gradually clarified [40–42]. The efficacy of several mAbs and small molecular blockers targeting CD47 has been tested in clinical practice [15,16,43]. Along with the progress, both mAb and nanobody probes targeting immune checkpoints (e.g., PD-L1) have shown encouraging potential for patient screening and prognosis prediction [18,44]. Although radiolabeled mAbs may demonstrate the feasibility for imaging of CD47, the relatively large size of mAbs results in long blood half-lives and the use of long-lived radionuclides such as ^89^Zr and ^64^Cu. Additionally, the poor tumor penetration and relatively increased immunogenicity of mAb probes are inevitable in some cases. In contrast, nanobodies with small size, high stability, strong antigen-binding affinity, rapid tumor uptake, and tumor-penetrating ability are preferable carriers for molecular imaging and RIT.

To our knowledge, there are no studies on nanobody probes targeting CD47 so far. This raises the question of whether nanobody-derived immunoPET imaging can apply to CD47 as well. Therefore, on the basis of our previous work [34,35,38,45], we constructed a nanobody molecular imaging probe, [^68^Ga]Ga-NOTA-C2, which is very sensitive and accurate for detecting CD47 expression within the tumors. One drawback of the study was that we used 2 gamma γ-counters and the traditional γ-counter was not precisely calibrated, leading to differences between biodistribution data with ROI analysis results (Fig. 4). CD47 is overexpressed in a variety of tumors, and we only performed PET/CT imaging with [^68^Ga]Ga-NOTA-C2 in 3 models of colon cancer, ovarian cancer, and gastric cancer. Our unpublished data further showed excellent detection ability of [^68^Ga]Ga-NOTA-C2 and [^89^Zr]Zr-DFO-ABDC2 in pancreatic cancer with high expression of CD47. Since [^68^Ga]Ga-NOTA-C2 did not react with murine CD47, this tracer could not depict the expression patterns of CD47 in mouse tissues and organs. Our unpublished data showed that this tracer could also specifically identify CD47 expression in CD47-humanized models and blocking with ABDC2 could significantly decrease the CD47-mediated uptake. With the tracers in hand, we may potentially assess the CD47 dynamics before and after anti-CD47 therapies.

Because of the small molecular weight of the nanobodies, they are rapidly excreted through the kidneys when administered in vivo and partially retained in the kidneys. To improve the image quality, we used the method previously reported, such as injection of sodium maleate before tracer injection, to reduce renal retention [34]. The optimized imaging results showed a good target-to-background ratio. However, the clinical feasibility and safety profiles of those small compounds needed to be explored.

The short half-lives of nanobody probes, rapid renal clearance, and the resultant substantial kidney retention prevent further therapeutic uses of monovalent nanobodies. Fusing the targeting molecule to an albumin-binding moiety to increase the size of the construct by noncovalent binding to serum albumin is a useful strategy to extend in vivo half-life, improve the bioavailability, reduce the kidney and liver uptake, and increase the tumor uptake [38,46]. One variant denoted ABD035 (~5 kDa) displaying wild-type-like secondary structure content showed an apparent affinity for serum albumin in the femtomolar range [47]. In the current study, C2 was fused to ABD035 to improve biodistribution properties and pave the road for improved ^89^Zr-labeled immunoPET imaging and ^177^Lu-labeled RIT. Quantitative analysis of the [^89^Zr]Zr-DFO-ABDC2 immunoPET data revealed that ABD035 fusion significantly increased blood pool retention time and decreased radioactivity retention in the kidneys. After exploring the pharmacokinetics of ABDC2 in tumor-bearing mice using [^89^Zr]Zr-DFO-ABDC2, we labeled it with ^177^Lu for achieving CD47-targeted RIT. Preliminary experimental results showed that [^177^Lu]Lu-DOTA-ABDC2 RIT showed the potential to inhibit the growth of CD47-positive tumors. Nevertheless, this treatment modality is associated with unexpected toxicity along with good tumor suppression. To achieve a desirable result, we will use humanized mice for the following experiments and adjust the treatment protocols in terms of dosage and time intervals. In addition, ABD035 has an fM–pM level binding affinity with human or mouse serum albumin, which may cause excessive blood retention time and consequently cause unavoidable blood toxicity when the fusion protein was used as an RIT carrier. Currently, we are also using some other albumin-binding moieties with weaker affinity to balance the circulation time, therapeutic effect, and toxicity [48,49]. The ^177^Lu-labeling treatment experiment also initially validated the feasibility of ABDC2 as a therapeutic vector. RIT as an important treatment modality will be explored in our subsequent studies to achieve the desired treatment effect. In addition, we are developing nanobody-drug conjugates using ABDC2 as the targeting scaffold [37,50].

## Materials and Methods

### Tumor cell lines and flow cytometry

Two solid tumor cell lines, SKOV-3 and LS174T, were used in the experiment. The cell lines were purchased from the American Type Culture Collection (Manassas, VA, USA) and cultured in Dulbecco’s modified Eagle’s medium at 37 °C with 5% CO_2_. Also added to the media were 10% fetal bovine serum (GE Healthcare, Chicago, IL, USA) and 1% PenStrep (Invitrogen).

Flow cytometry was used to detect CD47 expression on the surface of SKOV-3 and LS174T cells. All the cells were washed with sterile PBS and stained with a purified mouse anti-human CD47 mAb (MCA-911, Clone BRIC126; Bio-Rad), followed by washing and incubation of 5 𝜇g/ml of Alexa Fluor 488-conjugated goat anti-mouse IgG (Jackson ImmunoResearch Laboratories). The second antibody-only group was only stained with Alexa Fluor 488-conjugated goat anti-mouse IgG. Cells after staining were examined on a MACSQuant cytometer (Miltenyi Biotec). The data and plots were analyzed by the FlowJo software.

### Expression and characterization of nanobodies and nanobody derivatives

A healthy alpaca was immunized with 2 mg of recombinant human CD47 protein (Cat: 12283-H08H; Sino Biological) mixed with an equal volume of Freund's complete adjuvant 6 times. Meanwhile, the extracellular domain of human CD47 fused to Fc (1 mg mixed with Freund's complete adjuvant) was used to immunize Xinjiang Bactrian camel 3 to 7 times. Phage display technology was used to obtain the positive clones, followed by next-generation sequencing of the selected clones. The sequences of 3 representative clones (C1 and C3 from Bactrian camel and C2 from alpaca) were cloned into pET-30a(+) and expressed in BL21(DE3) competent cells. Notably, N-terminal his tag (HHHHHH) and C-terminal Q tag ((GGGGS)_2_-LLQS) were inserted into the nanobody sequences for purification and site-specific conjugation, respectively. The details were provided in the supplemental data. ABDC2 was further developed by fusing C2 with ABD (ABD035) derived from the Streptococcal protein G with a flexible (G_4_S)_3_ linker and expressed in human embryonic kidney 293 cells [47].

### Chelator conjugation, radiometal labeling, and quality control

The detailed methods for chelator conjugation, ^68^Ga/^89^Zr/^177^Lu-labeling, and quality control of the tracers are provided in the supplemental file.

### Animal models, small animal imaging, and treatment studies

All animal experiments were conducted in compliance with the Institutional Animal Care and Use Committee (Renji Hospital, School of Medicine, Shanghai Jiao Tong University). Briefly, 1 × 10^6^ SKOV-3 and LS174T cells were suspended in sterile PBS and Matrigel matrix (Corning) with a volume ratio of 1:1 and then injected into the right posterior flanks of female Balb/c nude mice (4 to 5 wk; GemPharmatech). Tumor-free female Balb/c mice (GemPharmatech) aged 4 to 5 weeks were used to evaluate the circulation and excretion pathways of [^68^Ga]Ga-NOTA-C2 and [^68^Ga]Ga-NOTA-ABDC2. NCG mice (NOD-Prkdcem^26Cd52^Il2rgem^26Cd22^/Nju) were purchased from the National Model Animal Resource Information Platform (#T001475, Nanjing University, China) for establishing No. 490 gastric cancer PDX models. These models were used for immunoPET imaging 2 to 4 weeks after inoculation of the cells and tumor tissues, assessing the diagnostic or therapeutic efficacies of the developed nanobody probes.

The injection doses of [^68^Ga]Ga-NOTA-C2, [^68^Ga]Ga-NOTA-ABDC2, [^89^Zr]Zr-DFO-ABDC2, [^177^Lu]Lu-DOTA-C2, and [^177^Lu]Lu-DOTA-ABDC2 are summarized in Tables S1 and S2. The mice were anesthetized and placed in the prone position on the scanning bed. Mouse PET/CT imaging was performed using an IRIS PET/CT system (Inviscan Imaging Systems). The PET images were reconstructed using Monte-Carlo-based 3-dimensional ordered subset expectation maximization with ROIs drawn manually using the OsiriX Lite software (Pixmeo SARL). The ROI data presented as the percentage of injected dose per gram of tissue (%ID/g) were analyzed on PMOD (PMOD Technologies LLC) and Inveon Research Workplace (Siemens Preclinical Solutions). For the therapeutic studies, No. 490 PDX models were randomly divided into 5 groups (*n* = 5 for each group). The therapeutic intervention began 1 week after the inoculation of the tumors. Mice weights and tumor volumes were measured by a calibrated weight scale and vernier caliper every 2 d.

### Biodistribution and histopathological studies

The mice were sacrificed after PET imaging at the last time point. Samples including blood were collected and wet-weighed, and the radioactivity of the samples was counted using a traditional γ-counter (ZONKIA, GC-1200) and an automated γ-counter (PerkinElmer). The uptake value in terms of %ID/g (mean ± SD) was calculated and given for major organs or tissues. Please note that the gamma counting carried out on the traditional γ-counter was not as accurate as that detected by the recently installed PerkinElmer γ-counter.

Hematoxylin and eosin (H&E) and IHC staining of the fixed tissues were carried out to evaluate the expression pattern of CD47. Briefly, sections of 10 μm were cut and stained for H&E and CD47 following the standard protocols. Two different antibodies (B6H12, sc-12730, Santa Cruz Biotechnology, Inc.; HPA044659, polyclonal rabbit IgG, Atlas Antibodies) were used for IHC studies with the dilution rate of 1:100 and 1:500, respectively. All the stained tissues were scanned for subsequent analysis.

### Statistical analysis

Results are presented as mean value ± SD. Statistical analyses were carried out by the Prism 8.3 statistical software (GraphPad Software Inc.). Statistical significance for tumor volume between groups was determined by multiple t-tests, and a *P* value of less than 0.05 was statistically significant. (★*P* < 0.05, ★★*P* < 0.005, ★★★*P* < 0.0005 and ★★★★*P* < 0.0001).

## Data Availability

The data used to support the findings of this study are available from the corresponding author upon request.

## Supplementary Materials

Supplementary 1 Supplementary Methods. Fig. S1. Immunohistochemical images of CD47-weakly positive or negative tumors with B6H12 as the primary antibody. Fig. S2. SDS-PAGE (A) and Western blot (B) analysis for C1 cloned in pET-30a(+) and expressed in BL21(DE3) strain. Fig. S3. SDS-PAGE (A) and Western blot (B) analysis for C3 cloned in pET-30a(+) and expressed in BL21(DE3) strain. Fig. S4. SDS-PAGE analysis for C1. Lane M: Protein marker; Lane BSA: 2.00 μg; Lane R: Reducing condition. Fig. S5. SDS-PAGE (A) and Western blot (B) analysis for C2 cloned in pET-30a(+) and expressed in BL21(DE3) strain. Fig. S6. SDS-PAGE analysis for C2 (A) and ABDC2 (B). Fig. S7. Surface plasmon resonance (SPR) studies showing the affinity/kinetics of NOTA-C2 (A), NOTA-ABDC2 (B), DOTA-C2 (C), DOTA-ABDC2 (D), and DFO-ABDC2 (E) interacting with recombinant human CD47 protein, respectively. Fig. S8. Isotopic dilution experiment showing the dependency between labeling yield and Ga(NO_3_)_3_ specific activity. Fig. S9. Assessment of the radiochemical purity. Fig. S10. Assessment of the radiochemical yield (A) and radiochemical purity (B). Fig. S11. Assessment of the radiochemical purity. Fig. S12. [^68^Ga]Ga-NOTA-C2 (6.98 ± 1.38 MBq, *n* = 3) (A) and [^68^Ga]Ga-NOTA-ABDC2 (5.75 ± 0.29 MBq, *n* = 4) (B) immunoPET imaging in Balb/c mice 2 and 4 h after injection of the tracer. Fig. S13. Analysis of ROI (A) and biodistribution data (B) of [^68^Ga]Ga-NOTA-C2 immunoPET imaging in cell- and patient-derived models. Fig. S14. Hematoxylin and eosin (H&E) and immunohistochemistry staining of the resected tumors. Fig. S15. Optimized [^68^Ga]Ga-NOTA-C2 immunoPET imaging in SKOV-3 cancer models. Fig. S16. (A) The maximum intensity projection images fused with CT images of all the time points fairly showed the overall distribution and uptake of [^89^Zr]Zr-DFO-ABDC2 in No. 490 gastric PDX model across a week. Table S1. The characteristics of precursor for ^68^Ga and ^89^Zr labeling and dosage injected for different models of immunoPET imaging. Table S2. The groups’ details of initial [^177^Lu]Lu-DOTA-ABDC2 theranostics in gastric PDX models. Table S3. Dosimetric calculations.
