# Supplementary material for: Development and Characterization of Nanobody-Derived CD47 Theranostic Pairs in Solid Tumors
Source: Research (Wash D C). 2023 Mar 15;6:0077. doi: 10.34133/research.0077 (PMC10017100; doi:10.34133/research.0077)
Supplement: Supplementary 1 — Supplementary Methods. Fig. S1. Immunohistochemical images of CD47-weakly positive or negative tumors with B6H12 as the primary antibody. Fig. S2. SDS-PAGE (A) and Western blot (B) analysis for C1 cloned in pET-30a(+) and expressed in BL21(DE3) strain. Fig. S3. SDS-PAGE (A) and Western blot (B) analysis for C3 cloned in pET-30a(+) and expressed in BL21(DE3) strain. Fig. S4. SDS-PAGE analysis for C1. Lane M: Protein marker; Lane BSA: 2.00 μg; Lane R: Reducing condition. Fig. S5. SDS-PAGE (A) and Western blot (B) analysis for C2 cloned in pET-30a(+) and expressed in BL21(DE3) strain. Fig. S6. SDS-PAGE analysis for C2 (A) and ABDC2 (B). Fig. S7. Surface plasmon resonance (SPR) studies showing the affinity/kinetics of NOTA-C2 (A), NOTA-ABDC2 (B), DOTA-C2 (C), DOTA-ABDC2 (D), and DFO-ABDC2 (E) interacting with recombinant human CD47 protein, respectively. Fig. S8. Isotopic dilution experiment showing the dependency between labeling yield and Ga(NO3)3 specific activity. Fig. S9. Assessment of the radiochemical purity. Fig. S10. Assessment of the radiochemical yield (A) and radiochemical purity (B). Fig. S11. Assessment of the radiochemical purity. Fig. S12. [68Ga]Ga-NOTA-C2 (6.98 ± 1.38 MBq, n = 3) (A) and [68Ga]Ga-NOTA-ABDC2 (5.75 ± 0.29 MBq, n = 4) (B) immunoPET imaging in Balb/c mice 2 and 4 h after injection of the tracer. Fig. S13. Analysis of ROI (A) and biodistribution data (B) of [68Ga]Ga-NOTA-C2 immunoPET imaging in cell- and patient-derived models. Fig. S14. Hematoxylin and eosin (H&E) and immunohistochemistry staining of the resected tumors. Fig. S15. Optimized [68Ga]Ga-NOTA-C2 immunoPET imaging in SKOV-3 cancer models. Fig. S16. (A) The maximum intensity projection images fused with CT images of all the time points fairly showed the overall distribution and uptake of [89Zr]Zr-DFO-ABDC2 in No. 490 gastric PDX model across a week. Table S1. The characteristics of precursor for 68Ga and 89Zr labeling and dosage injected for different models of immunoPET imaging. [file research.0077.f1.docx]

**Title:** **Development and characterization of nanobody-derived CD47 theranostic pairs in solid tumors**

You Zhang^1, #^, Di Zhang^1, #^, Shuxian An^1^, Qiufang Liu^2^, Chenyi Liang^1^, Juan Li^3^, Ping Liu^4^, Changfeng Wu^5^, Gang Huang^1, *^, Weijun Wei^1,*^, Jianjun Liu^1,*^

^1^ Department of Nuclear Medicine, Institute of Clinical Nuclear Medicine, Renji Hospital, School of Medicine, Shanghai Jiao Tong University, Shanghai 200127, China.

^2^ Department of Nuclear Medicine, Fudan University Shanghai Cancer Center, Fudan University, Shanghai 200030, China.

^3^ Institute of Cancer and Basic Medicine, Chinese Academy of Sciences, The Cancer Hospital of the University of Chinese Academy of Sciences, Hangzhou 310022, Zhejiang, China.

^4^ School of Biomedical Engineering and Med-X Research Institute, Shanghai Jiao Tong University, Shanghai 200030, China.

^5^ Department of Biomedical Engineering, Southern University of Science and Technology, Shenzhen 518055, Guangdong, China.

^#^ The authors contributed equally to the work.

**^*^ Corresponding Authors:**

**Prof. Weijun Wei**,

Department of Nuclear Medicine, Renji Hospital, School of Medicine, Shanghai Jiao Tong University, Shanghai 200127, China; e-mail: [wwei@shsmu.edu.cn](mailto:wwei@shsmu.edu.cn); lab website (<https://www.weiweijun.com>).

**Prof. Gang Huang**

Department of Nuclear Medicine, Renji Hospital, School of Medicine, Shanghai Jiao Tong University, Shanghai 200127, China; e-mail: [huanggang@sumhs.edu.cn](mailto:huanggang@sumhs.edu.cn)

**Prof. Jianjun Liu**

Department of Nuclear Medicine, Renji Hospital, School of Medicine, Shanghai Jiao Tong University, Shanghai 200127, China; e-mail: [nuclearj@163.com](mailto:nuclearj@163.com).

**Supplementary materials**

**Supplementary methods**

**NOTA conjugation and ^68^Ga-labeling of C2 and ABDC2**

The experimental method of NOTA conjugation is as follows: dissolve 1−2 mg C2/ABDC2 in phosphate buffered saline (PBS), and adjust the pH of the solution with 0.1 M Na_2_CO_3_ (pH = 11.4) buffer to 9.0–10. Then dissolve freshly *p*-SCN-Bn-NOTA (CAS Number: 147597-66-8; Macrocyclics) in dimethyl sulfoxide (DMSO). Add the NOTA solution to the protein solution with a molar ratio of *p*-SCN-Bn-NOTA/protein (C2 or ABDC2) = 10:1. Shake the reaction system slowly at room temperature for 2 hours, then purify the coupled protein with a pre-equilibrated PD-10 desalting column (GE Healthcare) with PBS as the mobile phase. At last, an ultrafiltration tube (Merck Millipore) with a cut off value of 10 kD was employed to concentrate the coupled protein solution, and the concentration of conjugated nanobody was determined with a Nanodrop. Divide the product for subsequent experiment uses.

The number of chelators per C2 or ABDC2 was elucidated in a modified isotopic dilution experiment [1, 2]. Briefly, a fixed amount of NOTA-C2 or NOTA-ABDC2 (1 nmol) was placed into 1.5 mL plastic Eppendorf tubes containing 250 µL mixed solution (pH = 4.0–4.5) of 0.05 M sodium acetate (pH = 7) and ~3.7 MBq (100 μCi) of ^68^GaCl_3_. Following, vials were spiked with 0.1, 0.2, 0.5, 1.0 or 2.0 nmol of Ga(NO_3_)_3_ and the mixtures incubated at 37 °C for 10 min. Radiochemical yield, as determined by instant thin-layer chromatography (iTLC; Eckert & Ziegler Radiopharma Inc) was plotted against Ga(NO_3_)_3_ concentration, allowing for the moles required for 50% (N50) labeling to be calculated. The number of chelators per C2 or ABDC2 was computed by dividing N50 by twice the moles of NOTA-conjugates. The results showed an approximate 1.3 and 6.6 NOTA chelators per molecule of NOTA-C2 and NOTA-ABDC2, respectively (Supplementary Figure 15).

The gallium-68 labeling follows our previously reported protocol [3]. Briefly, 296–350 MBq of freshly eluted ^68^Ga in 0.05 M hydrogen chloride (pH = 1) was mixed with 100 μL of 0.05 M sodium acetate (pH = 7). The radiometal solution with a final volume of 2.1 mL (pH = 4.0–4.5) was added to 200–800μg of NOTA-C2/NOTA-ABDC2, followed by incubation of the mixture at 37 °C for 5–10 min under constant shaking (600 rpm). The final radiopharmaceuticals were purified using pre-equilibrated PD-10 columns and the radiochemical purity was assessed by instant thin-layer chromatography (iTLC; Eckert & Ziegler Radiopharma Inc). More specifically, 10 μL of the purified products were spotted into silica-impregnated radio iTLC plates, run with 0.1 M sodium citrate (pH = 4.5), and assessed by a calibrated iTLC. Radiolabeled tracers remained at the origin (Rf = 0.15) while free ^68^Ga moved with the solvent front (Rf = 0.8).

**DFO conjugation and ^89^Zr-labeling of ABDC2**

DFO conjugation and ^89^Zr labeling mainly refer to a classic literature [4]. Briefly, dissolve 3 mg ABDC2 in phosphate buffered saline (PBS), and adjust the pH of the solution with 0.1 M Na_2_CO_3_ buffer (pH = 11.4) to 8.9–9.1. Then dissolve freshly DFO (CAS Number: 1222468-90-7; Macrocyclics) in DMSO. Add the DFO solution to the protein solution with a molar ratio of DFO/ABDC2 = 5:1. Shake the reaction system slowly at room temperature for thirty minutes, then purify the coupled protein with a pre-equilibrated PD-10 desalting column (GE Healthcare) with PBS as the mobile phase. At last, an ultrafiltration tube (Merck Millipore) with a cut off value of 10 kD was employed to concentrate the coupled protein solution, and the concentration of conjugated nanobody was determined with a NanoDrop. Divide the product for labeling studies. Zirconium-89 was freshly produced at our department. For ^89^Zr-labeling, the [^89^Zr]Zr-oxalic acid solution (450 µl, 100 MBq) was mixed with 1 M Na_2_CO_3_ buffer solution (400 µl), resulting in a final pH = 7 of the reaction. Then pipette successively 500 µl of 0.5 M HEPES (pH 7.1–7.3), 200 µl of DFO-ABDC2 (360 µg) into the reaction vial. Incubate for 1 h at room temperature while gently shaking the reaction vial. The final radiopharmaceuticals were purified using pre-equilibrated PD-10 columns and the radiochemical purity was assessed by iTLC. iTLC quality control was consistent with the method described above. It should be pointed out that, as shown in Supplementary Figure 8, the Rf values of (a) and (b) are different because of the different starting positions of the iTLC plates during the iTLC experiments of [^89^Zr]Zr-DFO-ABDC2.

**DOTA conjugation and 177Lu-labeling of C2 and ABDC2**

For ^177^Lu-labeling of C2 and ABDC2, we chose *p*-SCN-Bn-DOTA (CAS Number: 127985-74-4; Macrocyclics) as the chelator and the conjugation strategy was similar to the above-mentioned protocols. The ^177^Lu-labeling protocol was similar to that reported by Cheung et al. [5, 6].

**Supplementary figures and figure captions**


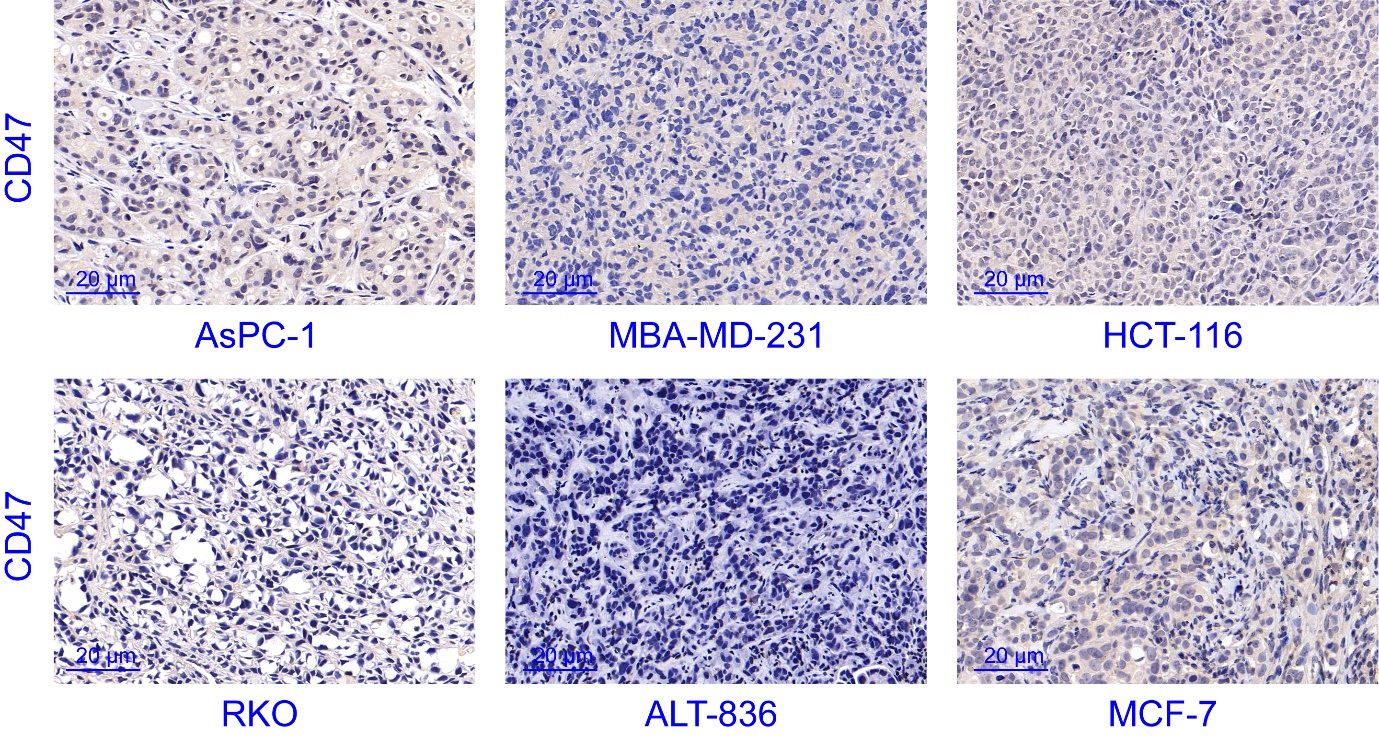


**Supplementary Figure 1.** Immunohistochemical images of CD47-weakly positive or negative tumors with B6H12 as the primary antibody.


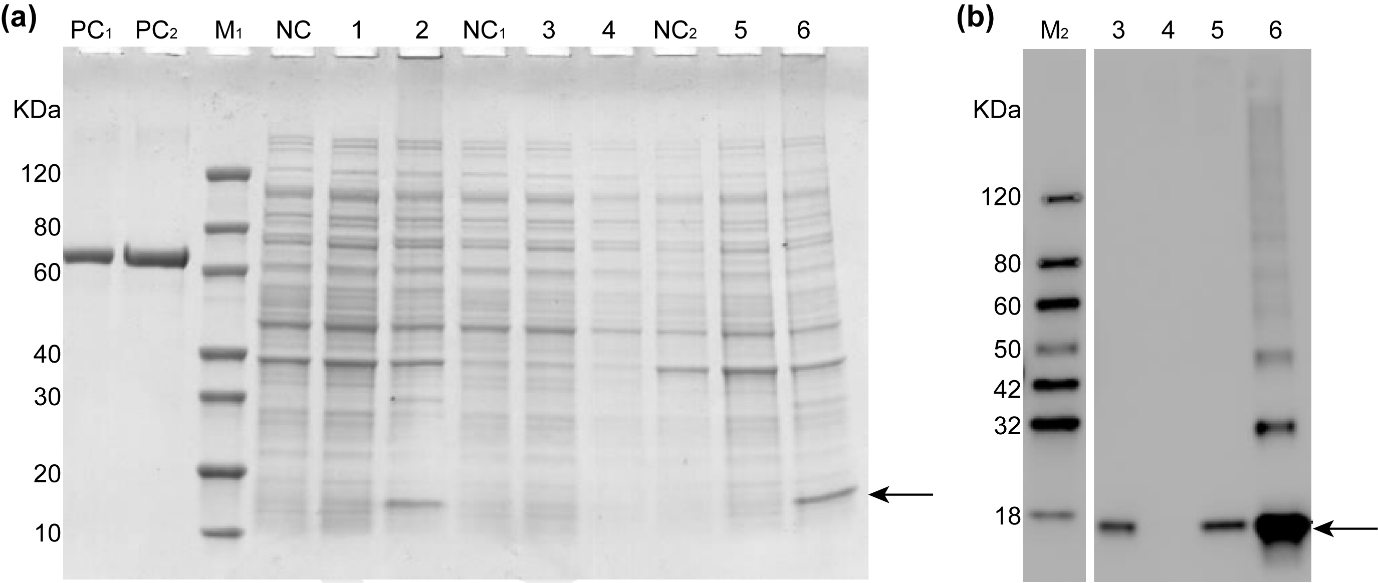


**Supplementary Figure 2.** SDS-PAGE (a) and western blot (b) analysis for C1 cloned in pET-30a(+) and expressed in BL21(DE3) strain. Lane M_1_: Protein marker; Lane M_2_: Western blot marker; Lane PC_1_: BSA (1 µg); Lane PC_2_: BSA (2 µg); Lane NC: Cell lysate without induction; Lane 1: cell lysate with induction for 16 h at 15 ℃; Lane 2: cell lysate with induction for 4 h at 37 ℃; Lane NC_1_: Supernatant of cell lysate without induction; Lane 3: Supernatant of cell lysate with induction for 16 h at 15 ℃; Lane 4: Supernatant of cell lysate with induction for 4 h at 37 ℃; Lane NC_2_: Debris of cell lysate without induction; Lane 5: Debris of cell lysate with induction for 16 h at 15 ℃; Lane 6: Debris of cell lysate with induction for 4 h at 37 ℃. The primary antibody for western blot is the anti-His antibody (GenScript, Cat. No. A00186).


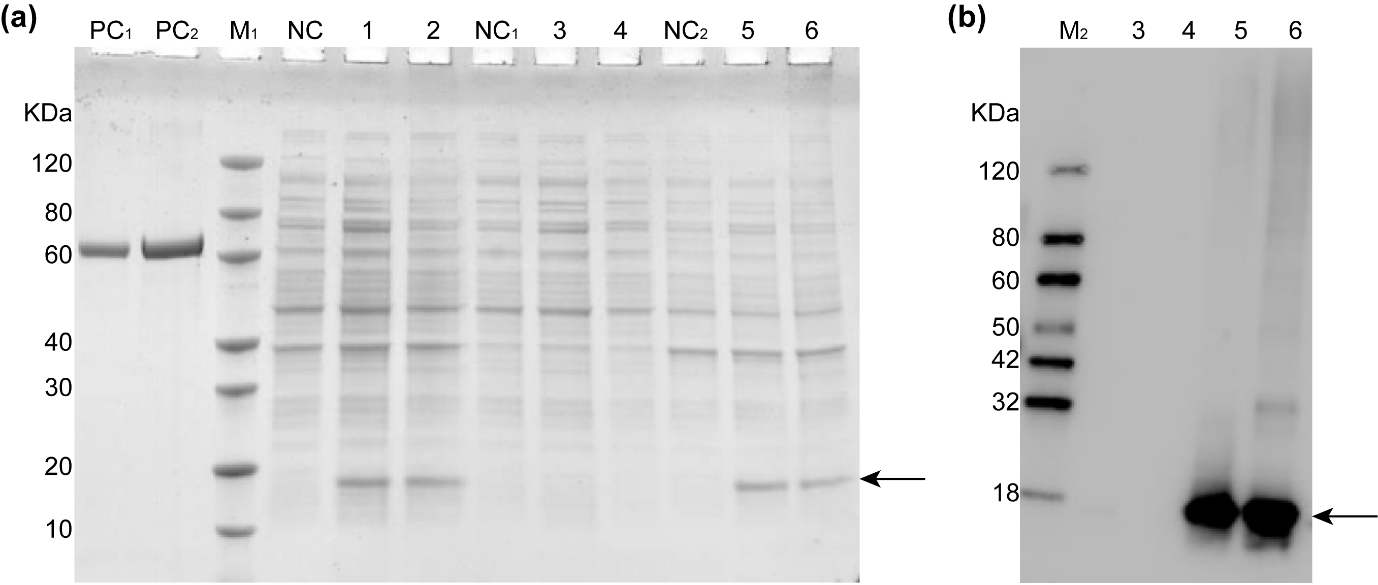


**Supplementary Figure 3.** SDS-PAGE (a) and western blot (b) analysis for C3 cloned in pET-30a(+) and expressed in BL21(DE3) strain. Lane M_1_: Protein marker; Lane M_2_: Western blot marker; Lane PC_1_: BSA (1 µg); Lane PC_2_: BSA (2 µg); Lane NC: Cell lysate without induction; Lane 1: cell lysate with induction for 16 h at 15 ℃; Lane 2: cell lysate with induction for 4 h at 37 ℃; Lane NC_1_: Supernatant of cell lysate without induction; Lane 3: Supernatant of cell lysate with induction for 16 h at 15 ℃; Lane 4: Supernatant of cell lysate with induction for 4 h at 37 ℃; Lane NC_2_: Debris of cell lysate without induction; Lane 5: Debris of cell lysate with induction for 16 h at 15 ℃; Lane 6: Debris of cell lysate with induction for 4 h at 37 ℃. The primary antibody for western blot is the anti-His antibody (GenScript, Cat. No. A00186).

**
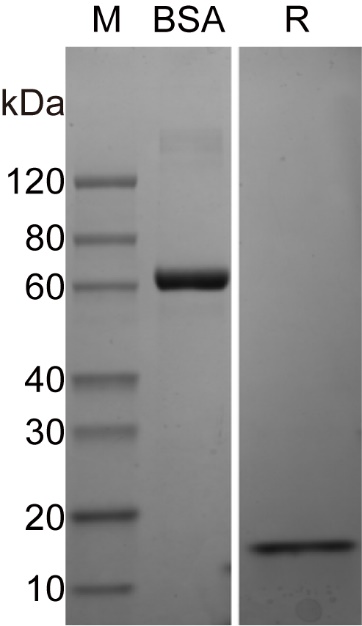
**

**Supplementary Figure 4.** SDS-PAGE analysis for C1. Lane M: Protein marker; Lane BSA: 2.00 µg; Lane R: Reducing condition.

**
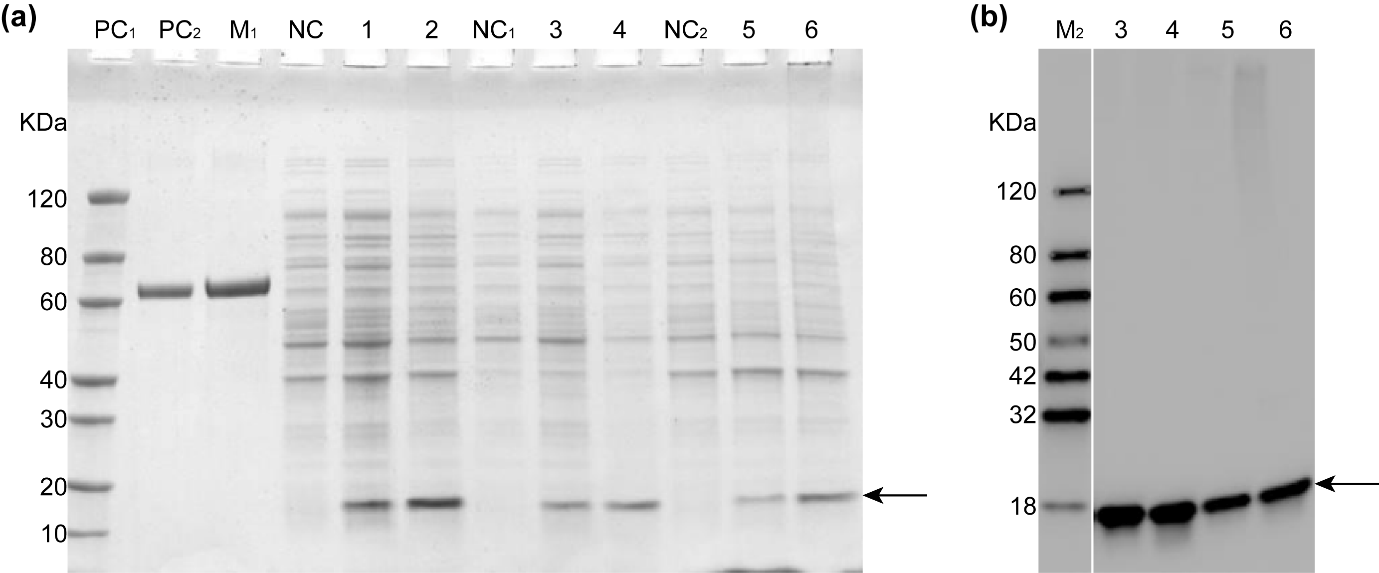
**

**Supplementary Figure 5.** SDS-PAGE (a) and western blot (b) analysis for C2 cloned in pET-30a(+) and expressed in BL21(DE3) strain. Lane M_1_: Protein marker; Lane M_2_: Western blot marker; Lane PC_1_: BSA (1 µg); Lane PC_2_: BSA (2 µg); Lane NC: Cell lysate without induction; Lane 1: cell lysate with induction for 16 h at 15 ℃; Lane 2: cell lysate with induction for 4 h at 37 ℃; Lane NC_1_: Supernatant of cell lysate without induction; Lane 3: Supernatant of cell lysate with induction for 16 h at 15 ℃; Lane 4: Supernatant of cell lysate with induction for 4 h at 37 ℃; Lane NC_2_: Debris of cell lysate without induction; Lane 5: Debris of cell lysate with induction for 16 h at 15 ℃; Lane 6: Debris of cell lysate with induction for 4 h at 37 ℃. The primary antibody for western blot is the anti-His antibody (GenScript, Cat. No. A00186).


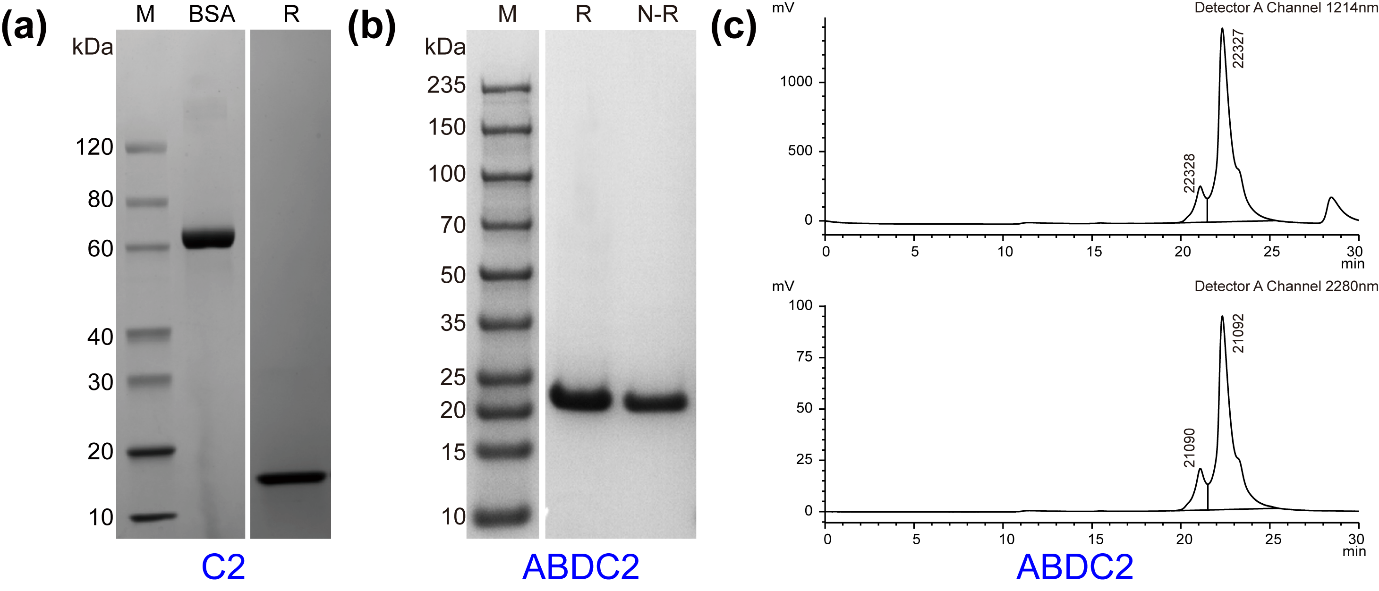


**Supplementary Figure 6.** SDS-PAGE analysis for C2 (a) and ABDC2 (b). Lane M: Protein marker; Lane BSA: 2.00 µg; Lane R: Reducing condition. **(c)** Characterization of ABDC2 by high-performance liquid chromatography (HPLC).


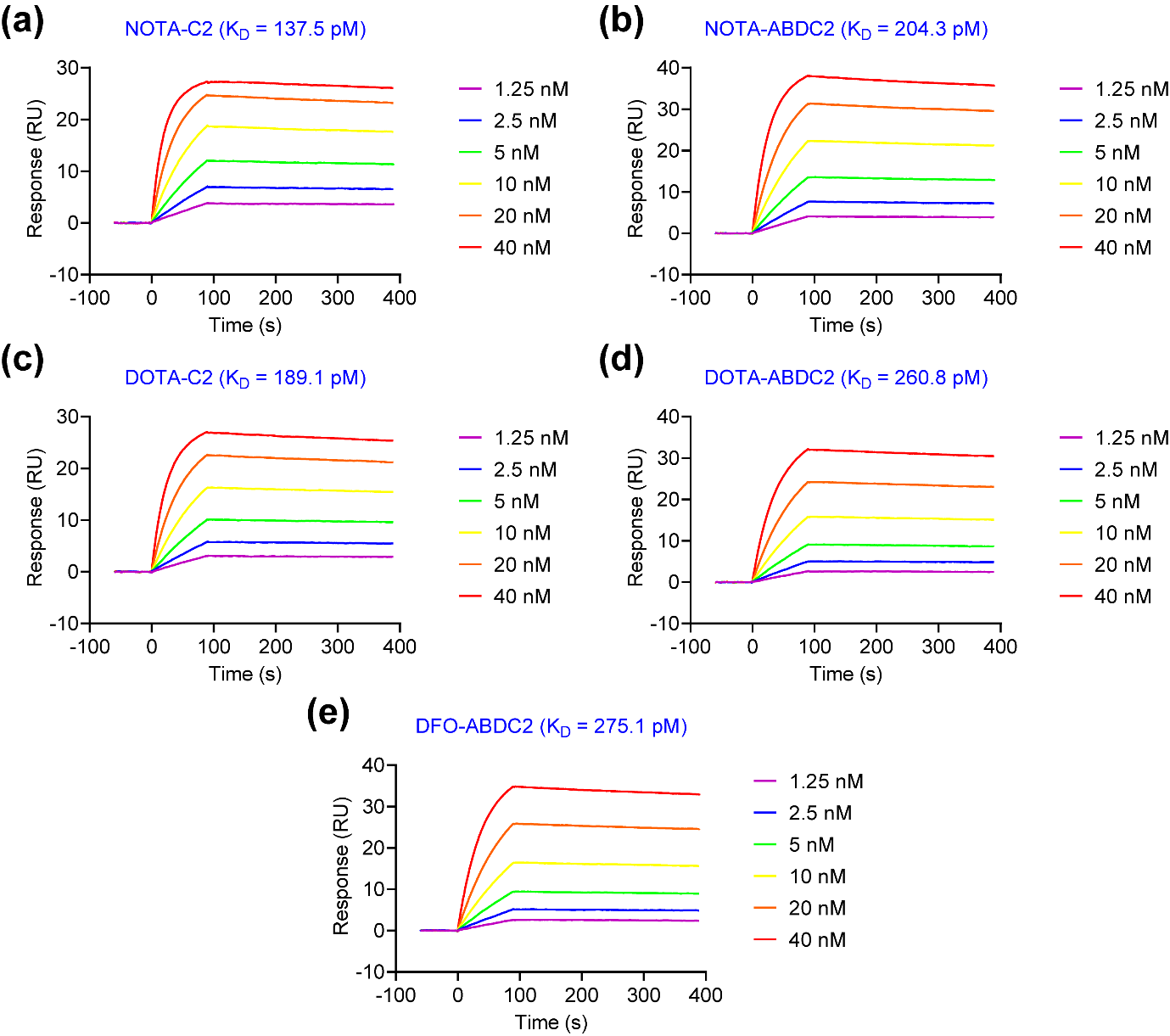


**Supplementary Figure 7.** Surface plasmon resonance (SPR) studies showing the affinity/kinetics of NOTA-C2 (a), NOTA-ABDC2 (b), DOTA-C2 (c), DOTA-ABDC2 (d) and DFO-ABDC2 (e) interacting with recombinant human CD47 protein, respectively.


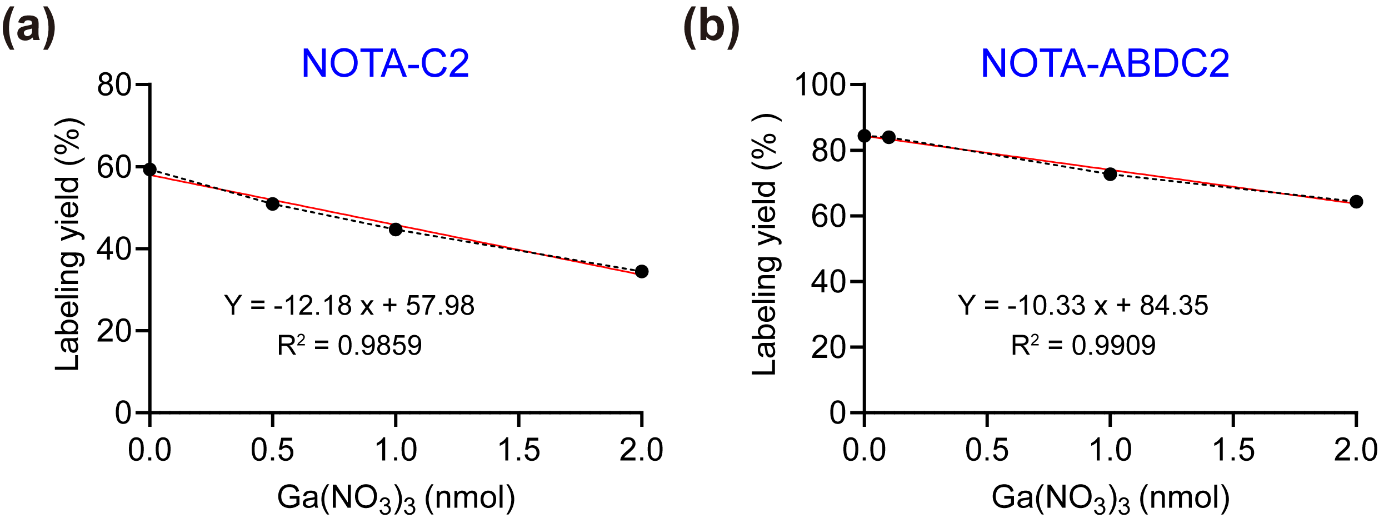


**Supplementary Figure 8.** Isotopic dilution experiment showing the dependency between labeling yield and Ga(NO_3_)_3_ specific activity. The estimated number of NOTA per C2 or ABDC2 was 1.3 (a) and 6.6 (b), repectively.


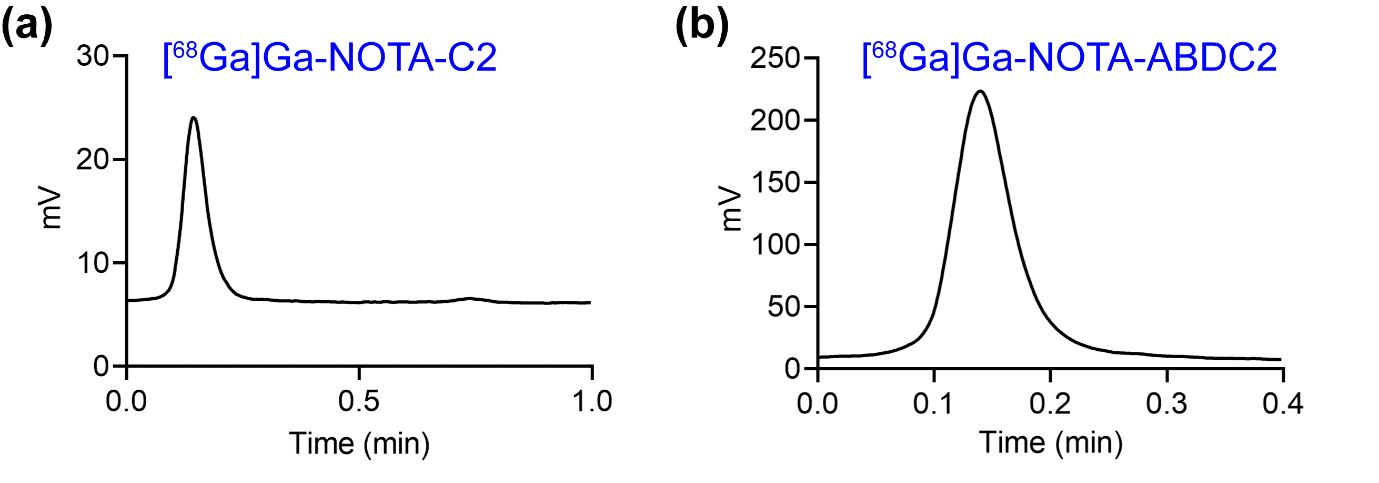


**Supplementary Figure 9**. Assessment of the radiochemical purity. [^68^Ga]Ga-NOTA-C2 (Rf = 0.15) (a) and [^68^Ga]Ga-NOTA-ABDC2 (Rf = 0.15) (b) remained intact immediately at purification in the PBS buffer as assessed by instant thin-layer chromatography.


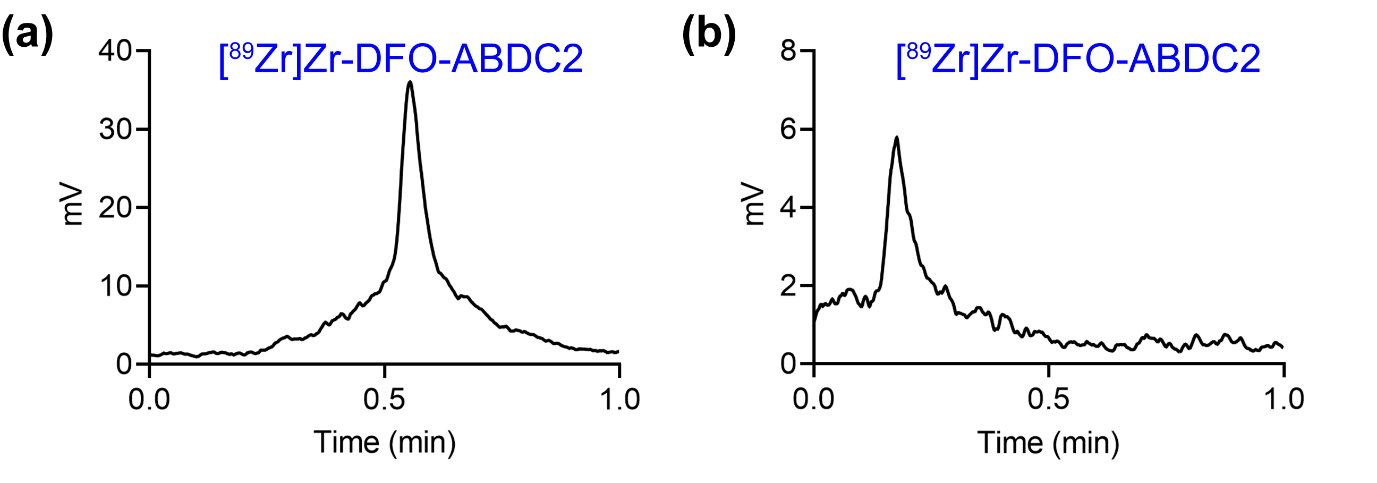


**Supplementary Figure 10**. Assessment of the radiochemical yield (a) and radiochemical purity (b). [^89^Zr]Zr-DFO-ABDC2 remained intact immediately at purification in the PBS buffer as assessed by instant thin-layer chromatography.


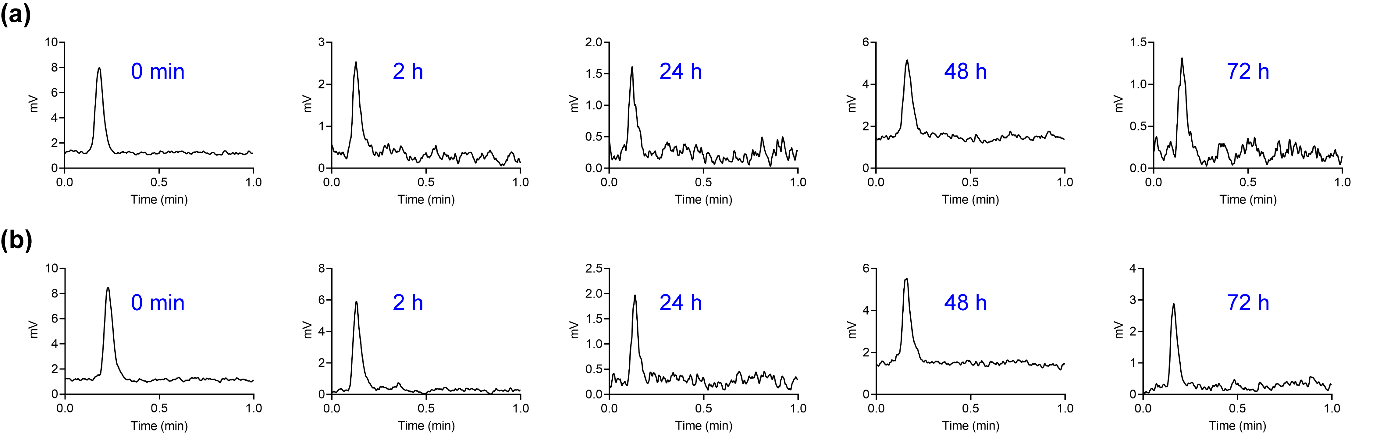


**Supplementary Figure 11**. Assessment of the radiochemical purity. [^177^Lu]Lu-DOTA-C2 (a) and [^177^Lu]Lu-DOTA-ABDC2 (b) remained intact within 72 h at purification in the PBS buffer as assessed by thin-layer chromatography.


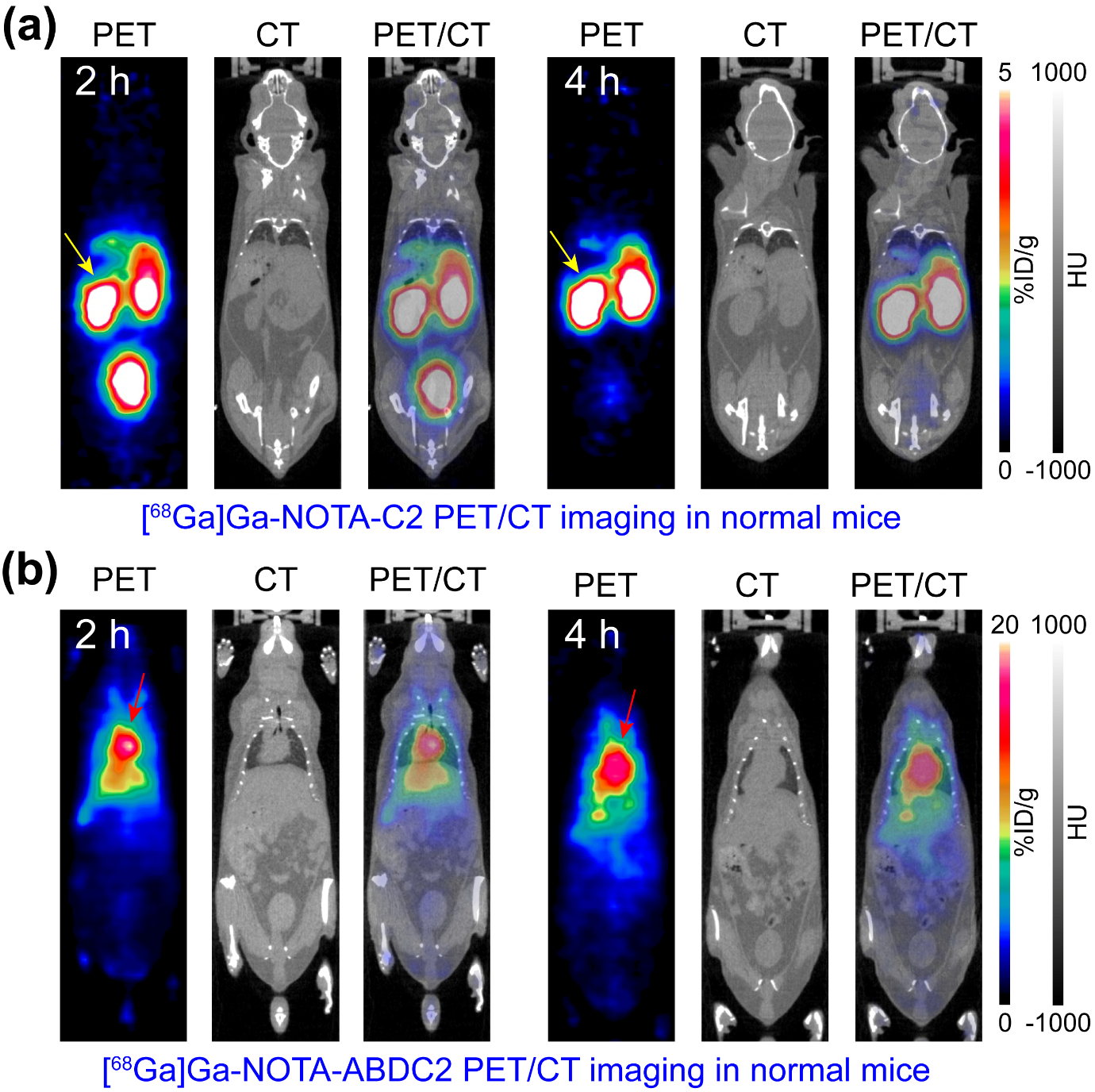


**Supplementary Figure 12**. [^68^Ga]Ga-NOTA-C2 (6.98 ± 1.38 MBq, n = 3) (a) and [^68^Ga]Ga-NOTA-ABDC2 (5.75 ± 0.29 MBq, n = 4) (b) immunoPET imaging in Balb/c mice 2 h and 4 h after injection of the tracer. Coronal images at different slices showed clear delineation of the kidneys (yellow arrows) and hearts (red arrows).


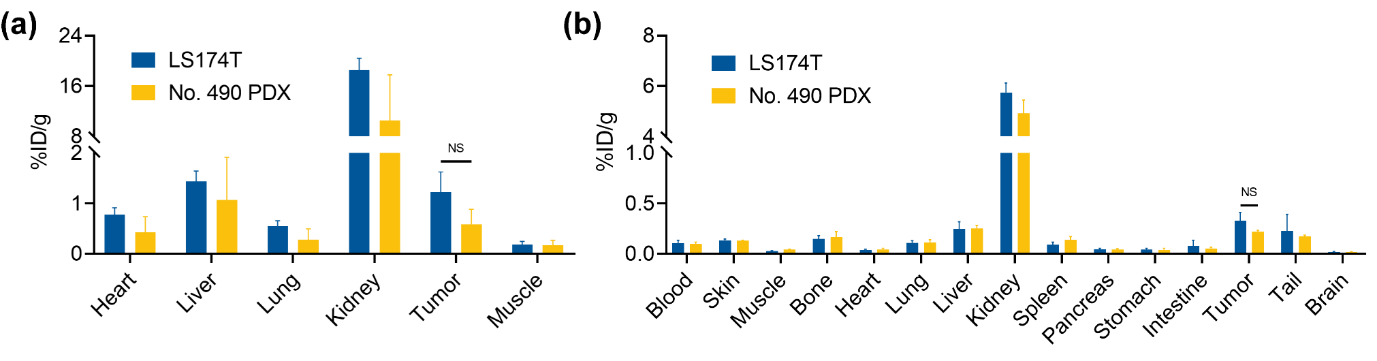


**Supplementary Figure 13.** Analysis of ROI (a) and biodistribution data (b) of [^68^Ga]Ga-NOTA-C2 immunoPET imaging in cell- and patient-derived models.


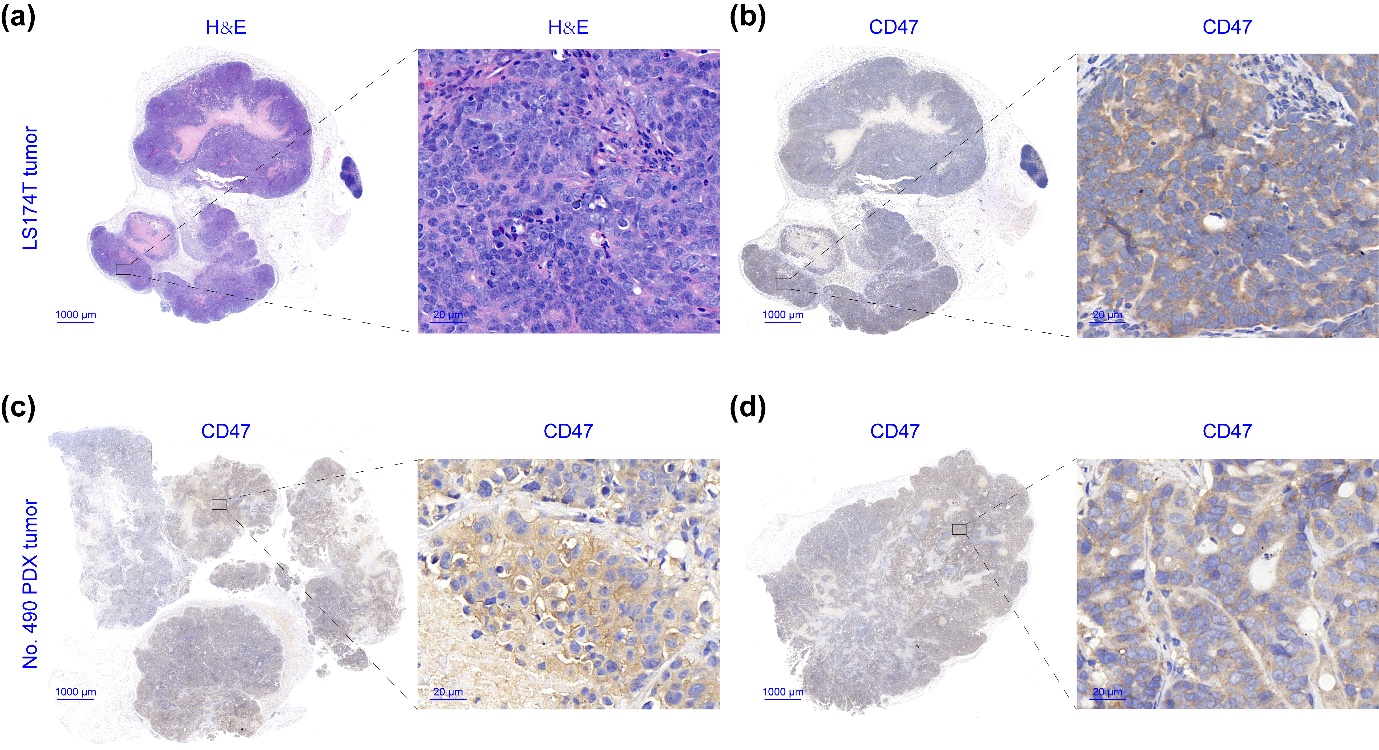


**Supplementary Figure 14**. Hematoxylin and eosin (H&E) and immunohistochemistry staining of the resected tumors. (a, b) H&E staining (a) and CD47 antigen immunohistochemical staining (b) of LS174T tumors with B6H12. (c, d) The CD47 antigen immunohistochemical staining of No. 490 PDX tumors with B6H12.

**
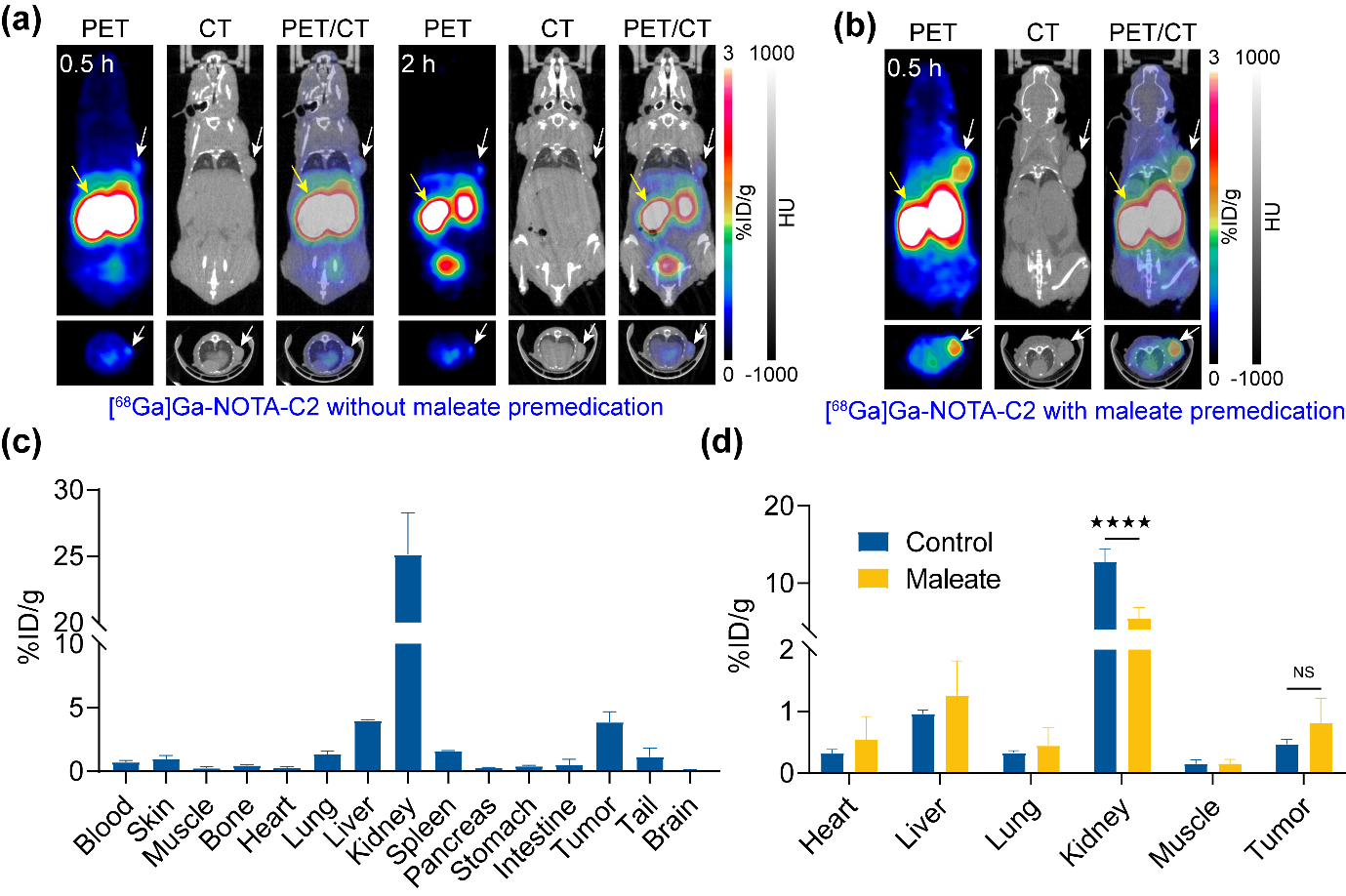
**

**Supplementary Figure 15.** Optimized [^68^Ga]Ga-NOTA-C2 immunoPET imaging in SKOV-3 cancer models. Representative coronal (up panels) and axial (down panels) [^68^Ga]Ga-NOTA-C2 immunoPET/CT images of the SKOV-3−bearing mice in the (a) control group and (b) maleate intervention group. Coronal and axial images showed clear delineation of the tumors (white arrows) and kidneys (yellow arrows). (c) *Ex vivo* biodistribution data showing detailed uptake of [^68^Ga]Ga-NOTA-C2 in the tumor, blood, major organs, and tissues. (d) ROI analysis of [^68^Ga]Ga-NOTA-C2 in the control group and maleate intervention group at 0.5 h after tracer injection. ★★★★: P < 0.0001.


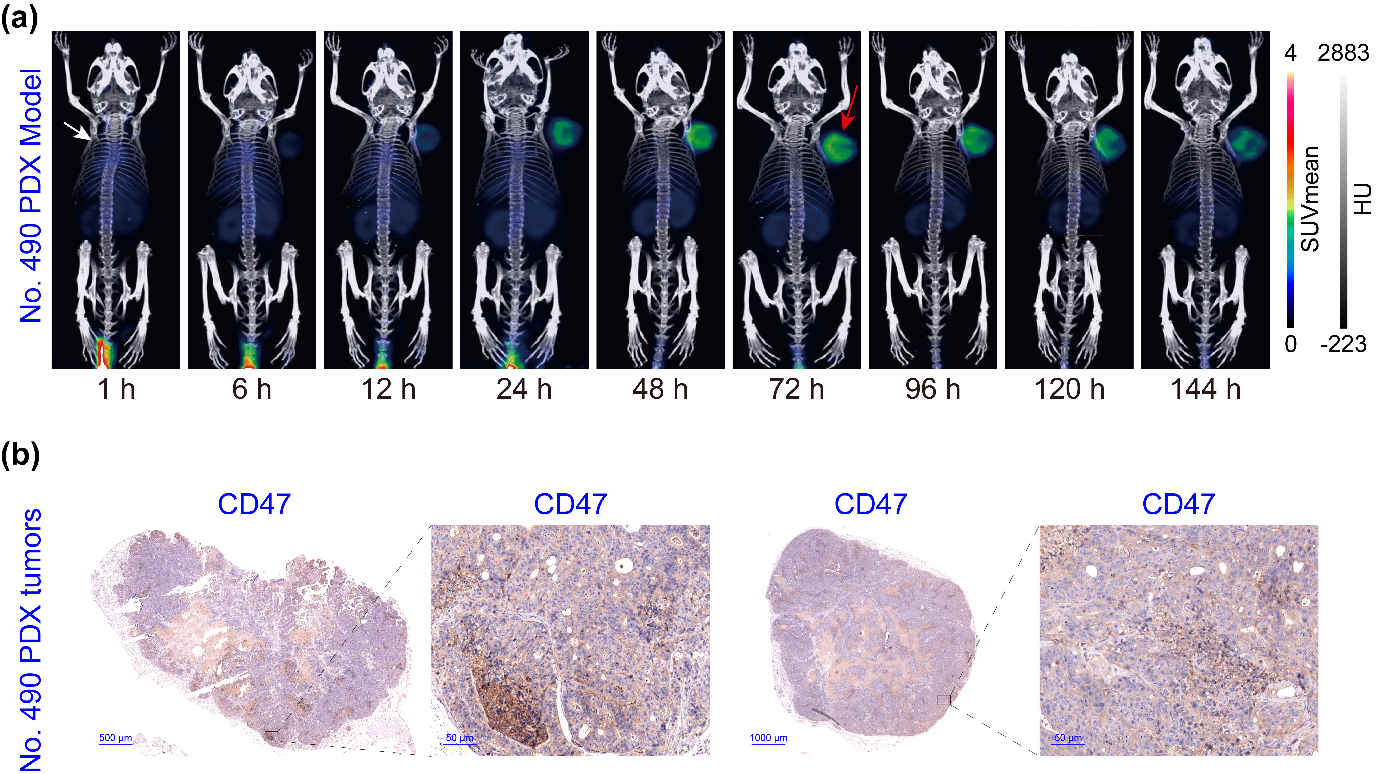


**Supplementary Figure 16.** (a) The maximum intensity projection images fused with CT images of all the time points fairly showed the overall distribution and uptake of [^89^Zr]Zr-DFO-ABDC2 in No. 490 gastric PDX model across a week. (b) The CD47 antigen immunohistochemical staining of No. 490 PDX tumors with HPA044659.

| **Probe** | **Precursor** | **The Mass of the precursor** | **Radion-uclides** | **The activity of eluted Radionuclides incubated with precursor (MBq)** | **Molar activity**  **(mCi/umol)** | **Mice Modes** | **Injection Dose (MBq)** |
| --- | --- | --- | --- | --- | --- | --- | --- |
| [^68^Ga]Ga-NOTA-C2 | NOTA-C2 | 812.45 µg | ^68^Ga | 327.08 | 18.9 | normal Balb/c mice | 6.98 ± 1.38, n = 3 |
| [^68^Ga]Ga-NOTA-ABDC2 | NOTA-ABDC2 | 331.7 µg | ^68^Ga | 406.26 | 81.2 | normal Balb/c mice | 5.75 ± 0.29, n = 4 |
| [^68^Ga]Ga-NOTA-C2 | NOTA-C2 | 791.65 µg | ^68^Ga | 293.04 | 54.7 | SKOV-3 | 11.26 ± 2.84, n = 8 |
| [^68^Ga]Ga-NOTA-C2 | NOTA-C2 | 1.5 mg | ^68^Ga | 350.39 | 17.8 | LS174T | 9.10 ± 0.89, n = 3 |
|  |  |  |  |  |  | No. 490 PDX | 6.50 ± 0.46, n = 3 |
| [^68^Ga]Ga-NOTA-ABDC2 | NOTA-ABDC2 | 331.7 µg | ^68^Ga | 358.9 | 58.3 | No. 490 PDX | 4.31 ± 0.22, n = 4 |
| [^89^Zr]Zr-DFO-ABDC2 | DFO-ABDC2 | 360.2 µg | ^89^Zr | 34.41 | 34.6 | No. 490 PDX | 4.10 ± 0.50, n = 3 |

**Supplementary Table 1.** The characteristics of precursor for ^68^Ga and ^89^Zr labeling and dosage injected for different models of immunoPET imaging.

**Supplementary Table 2.** The groups’ details of initial [^177^Lu]Lu-DOTA-ABDC2 theranostics in gastric PDX models

| **Group** | **Intervention** | **Radioactivity** | **Volume (µl)** |
| --- | --- | --- | --- |
| Negative control | PBS, n = 5 | ╳ | 200 |
| [^177^Lu]Lu-DOTA-C2 | [^177^Lu]Lu-DOTA-C2 | 6.66 ± 0.80MBq, n = 5 | 200 |
| [^177^Lu]Lu-DOTA-ABDC2 (Low dose) | [^177^Lu]Lu-DOTA-ABDC2 | 7.07 ± 0.98MBq, n = 5 | 200 |
| [^177^Lu]Lu-DOTA-ABDC2 (High dose) | [^177^Lu]Lu-DOTA-ABDC2 | 13.46 ± 0.63MBq, n = 5 | 200 |
| ABDC2 only | 500ug ABDC2, n = 5 | ╳ | 200 |

**Supplementary Table 3.** Dosimetric calculations

|  | **[^89^Zr]Zr-DFO-ABDC2** |
| --- | --- |
| **Tissue** | **AD (mGy/MBq)** |
| Spleen | 0.09 |
| Liver | 7.02 |
| Kidney | 0.36 |
| Pancreas | 0.12 |
| Tumor | 424.38 |

NOTE: Dosimetric calculations expressed in mGy per injected MBq for the spleen, liver, kidneys, pancreas, and tumor for ABDC2 labeled with the ^89^Zr. The absorbed dose (AD) calculated for normal tissues and tumors was based on NCG mouse ROI data, which were converted into adult human ADs. The calculated time-integrated activities were used as input in OLINDA/EXM using the adult male phantom for normal tissues, and using the sphere model to calculate the AD for the tumor.

**References:**

1. Hernandez R, Sun H, England C G, Valdovinos H F, Ehlerding E B, Barnhart T E, Yang Y, Cai W. CD146-targeted immunoPET and NIRF Imaging of Hepatocellular Carcinoma with a Dual-Labeled Monoclonal Antibody. *Theranostics.* 2016;6(11):1918-1933. <https://doi.org/10.7150/thno.15568>.

2. Meares C F, McCall M J, Reardan D T, Goodwin D A, Diamanti C I, McTigue M. Conjugation of antibodies with bifunctional chelating agents: isothiocyanate and bromoacetamide reagents, methods of analysis, and subsequent addition of metal ions. *Anal Biochem.* 1984;142(1):68-78. <https://doi.org/10.1016/0003-2697(84)90517-7>.

3. Wang C, Chen Y, Hou Y N, Liu Q, Zhang D, Zhao H, Zhang Y, An S, Li L, Hou J, et al. ImmunoPET imaging of multiple myeloma with [(68)Ga]Ga-NOTA-Nb1053. *Eur J Nucl Med Mol Imaging.* 2021;48(9):2749-2760. <https://doi.org/10.1007/s00259-021-05218-1>.

4. Vosjan M J, Perk L R, Visser G W, Budde M, Jurek P, Kiefer G E, van Dongen G A. Conjugation and radiolabeling of monoclonal antibodies with zirconium-89 for PET imaging using the bifunctional chelate p-isothiocyanatobenzyl-desferrioxamine. *Nat Protoc.* 2010;5(4):739-743. <https://doi.org/10.1038/nprot.2010.13>.

5. Santich B H, Cheal S M, Ahmed M, McDevitt M R, Ouerfelli O, Yang G, Veach D R, Fung E K, Patel M, Burnes Vargas D, et al. A Self-Assembling and Disassembling (SADA) Bispecific Antibody (BsAb) Platform for Curative Two-step Pretargeted Radioimmunotherapy. *Clin Cancer Res.* 2021;27(2):532-541. <https://doi.org/10.1158/1078-0432.Ccr-20-2150>.

6. Chandler C S, Bell M M, Chung S K, Veach D R, Fung E K, Punzalan B, Burnes Vargas D, Patel M, Xu H, Guo H F, et al. Intraperitoneal Pretargeted Radioimmunotherapy for Colorectal Peritoneal Carcinomatosis. *Mol Cancer Ther.* 2022;21(1):125-137. <https://doi.org/10.1158/1535-7163.Mct-21-0353>.
